# Development of Water‐Soluble 3‐Nitro‐2‐Pyridinesulfenate for Disulfide Bond Formation of Peptide Under Aqueous Conditions

**DOI:** 10.1002/chem.202500855

**Published:** 2025-05-03

**Authors:** Akihiro Taguchi, Megumi Sakata, Ryusei Yamamoto, Hayate Shida, Saeka Kuraishi, Sho Konno, Kentaro Takayama, Atsuhiko Taniguchi, Yoshio Hayashi

**Affiliations:** ^1^ Department of Medicinal Chemistry School of Pharmacy Tokyo University of Pharmacy and Life Sciences 1432‐1 Horinouchi Hachioji Tokyo 192–0392 Japan; ^2^ Laboratory of Medicinal Chemistry and Chemical Biology School of Life Sciences Tokyo University of Pharmacy and Life Sciences 1432‐1 Horinouchi Hachioji Tokyo 192–0392 Japan; ^3^ Laboratory of Environmental Biochemistry Kyoto Pharmaceutical University 5 Misasaginakauchi‐cho Yamashina Kyoto 607–8414 Japan

**Keywords:** 3‐nitro‐2‐pyridinesulfenate, disulfide bond, native chemical ligation, oxidation, peptides

## Abstract

Establishing an efficient method for the synthesis of disulfide bonds in peptides is an important challenge for the further development of several research fields, including peptide‐based medicinal chemistry and biochemistry. Herein, we report the development of a novel highly water‐soluble 3‐nitro‐2‐pyridine (Npy) sulfenate that efficiently forms intramolecular disulfide bonds in reduced peptides. Moreover, Npy‐sulfenate formed a disulfide bond in a thiol‐containing peptide prepared by thiol‐additive‐free native chemical ligation (NCL), as demonstrated by the one‐pot synthesis of adrenomedullin, which contains 52 amino acid residues. This study provides a new one‐pot synthetic methodology for preparing mid‐sized cyclic disulfide peptides via sulfenate‐mediated oxidation.

## Graphical abstract

Highly water‐soluble Npy‐sulfenate functions as an efficient oxidizing agent for the formation of disulfide bonds in thiol‐containing peptides. The Npy‐sulfenate can be used to form a disulfide bond with a thiol‐containing peptide produced by thiol‐additive‐free NCL, thereby facilitating the one‐pot synthesis of adrenomedullin. These results provide a new one‐pot synthetic methodology for cyclic disulfide peptides based on sulfenate‐mediated oxidation.

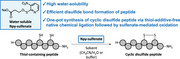

## 1 Introduction

Disulfide bonds in peptides rigidly stabilize their 3D structures, while also improving their affinities and selectivities for target molecules and their resistance to enzymatic digestion.^[^ ^1^ ^]^ During the past quarter‐century, disulfide bond–containing peptides, such as ziconotide, a potent and selective N‐type calcium channel blocker,^[^ ^2^ ^]^ and plecanatide, a guanylate cyclase C agonist,^[^ ^3^ ^]^ have been developed as therapeutic agents. Recently, motixafortide^[^ ^4^ ^]^ a selective chemokine receptor 4 (CXCR4) inhibitor and ^177^Lu‐dotatate^[^ ^5^ ^]^—the chelated complex formed between dotatate and the lutetium radioisotope and is used in peptide receptor radionuclide therapy—have been approved by the Food and Drug Administration in the United States. Furthermore, disulfide bonds play important crosslinking roles, such as in peptide–^[^ ^6^ ^]^ and antibody–drug conjugations^[^ ^7^ ^]^ composed of various functional molecules.

In general, disulfide‐containing peptides are synthesized in solution by treating a free or protected thiol groups (trityl, acetamidomethyl, Bn, *t*‐Bu, etc.) of a linear peptide prepared by 9‐fluorenylmethyloxycarbonyl (Fmoc)/*t*‐Bu solid‐phase peptide synthesis (SPPS) with an appropriate oxidizing agent. As representative oxidants, DMSO,^[^ ^8^ ^]^ potassium ferricyanide,^[^ ^9^ ^]^ hydrogen peroxide,^[^ ^10^ ^]^ glutathione,^[^ ^11^ ^]^ sulfuryl chloride,^[^ ^12^ ^]^ *N*‐chlorosuccinimide,^[^ ^13^ ^]^ disulfiram,^[^ ^14^ ^]^ iodine (I_2_),^[^ ^15^ ^]^ sulfoxide‐silyl chloride,^[^ ^16^ ^]^ and thallium(III) trifluoroacetate^[^ ^17^ ^]^ are available.

Disulfide bonds can also be formed using the 3‐nitro‐2‐pyridinesulfenyl (Npys) group developed by Matsueda et al. This Npys group not only functions as a stable protecting group against trifluoroacetic acid (TFA) in Cys side chains but also as an active disulfide that selectively and rapidly forms a disulfide bond with another unprotected thiol group.^[^ ^18^ ^]^ These reactivities of the Npys group contribute to the regioselective formation of intra‐ or intermolecular disulfide bonds of peptide.^[^ ^19^ ^]^ For example, as the great pioneer work, Moroder group used the Npys group to regioselectively form disulfide bonds across multi‐stranded polypeptides and successfully achieved the first synthesis of homo‐ and heterotrimeric collagen model peptides using an artificial cysteine knot.^[^ ^20^ ^]^ Furthermore, Chino et al. synthesized an antiparallel dimer of alpha human atrial natriuretic peptide (α‐hANP) in combination with Npys‐mediated disulfide bond formation and iodine oxidation.^[^ ^21^ ^]^ We developed a one‐pot solid‐phase disulfide ligation (SPDSL) protocol ^[^ ^22^ ^]^ that facilitates the efficient synthesis of disulfide‐linked heterodimers from two different thiol‐containing components using Npys‐Cl‐immobilized resin (Scheme S1A in the Supporting Information). In addition, as a synthetic methodology for the preparation of cyclic peptides that is conceptually different from the conventional method, we introduced disulfide‐driven cyclic peptide synthesis (DdCPS),^[^ ^23^ ^]^ which involves preparing a disulfide peptide by one‐pot SPDSL followed by intramolecular cyclization. This method enables intramolecular cyclization to occur highly regioselectively and more efficiently than intermolecular amide bond formation owing to the proximity of each reaction center via the prior construction of the disulfide bond between the two peptide fragments (Scheme S1B).

**Figure 1:**
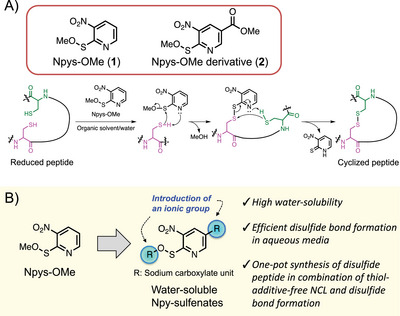
A) Plausible mechanism for the Npys‐OMe‐mediated disulfide bond formation in a thiol‐containing peptide. B) Molecular designs of Npy‐sulfenates bearing ionic groups.

**Figure 2:**
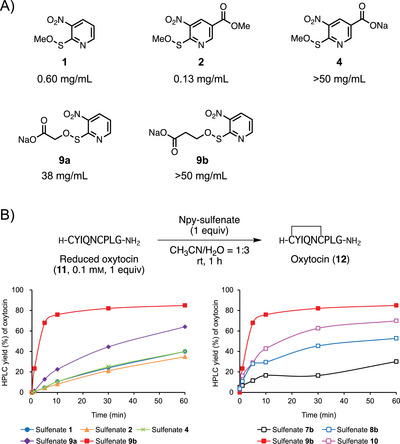
A) Evaluating the water solubilities of Npy‐sulfenates. The values are the means of three measurements. B) Npy sulfenate–mediated disulfide bond formation in reduced oxytocin (**11**).

We identified Npy‐sulfenate as a new Npys motif for disulfide bond formation of peptides. In particular, methyl 3‐nitro‐2‐pyridinesulfenate (**1**, Npys‐OMe) and its derivative **2** are physicochemically stable, mildly oxidizable, and induce the formation of intramolecular disulfide bonds from two free thiols at relatively high peptide concentrations under mildly acidic conditions.^[^ ^24^ ^]^ A plausible reaction mechanism is shown in Figure 1A,^[^ ^24a^ ^]^ in which an active disulfide would be formed via the interaction of the five‐membered ring between the thiol of the reduced peptide and Npys‐OMe (**1**). Disulfide exchange between the active disulfide and another thiol in the peptide would yield a cyclized peptide and 3‐nitropyridine‐2(1*H*)‐thione. The oxidation‐sensitive Trp, Met, and Tyr residues did not become involved in any side reactions during Npys‐OMe‐mediated disulfide formation.^[^ ^24a^ ^]^ We formed the disulfide bonds in monocyclic oxytocin and α‐hANP, and regioselective formed the disulfide bond in bicyclic α‐conotoxin ImI by exploiting the mild oxidative properties of Npys‐OMe. Our previous study revealed that Npy‐sulfenates **1** and **2** are promising oxidants for the formation of disulfide bonds of peptide. However, both compounds require the use of organic solvents (CH_3_CN and DMF) during disulfide bond formation of peptide owing to their low water solubilities (Figure 2A). Improving the water solubilities of these compounds is expected to facilitate the oxidation of two thiols to form an intramolecular disulfide in aqueous media, which may be applicable to efficiently forming disulfide‐bridged biomolecules, including proteins and antibodies that are prone to aggregation or denaturation in organic solvents.

In this study, we developed a novel highly water‐soluble Npy‐sulfenate that facilitates effective disulfide bond formation in aqueous media. This was achieved by designing and synthesizing an Npy‐sulfenate with an ionic sodium carboxylate group at the C‐5 position of the pyridine ring or at the methyl sulfenate moiety (Figure 1B). We evaluated the water solubilities of these synthetic sulfenates and demonstrated their disulfide bond‐forming potencies via a model study based on the synthesis of oxytocin. Moreover, we successfully combined sulfenate‐mediated disulfide bond formation with native chemical ligation (NCL),^[^ ^25^ ^]^ which resulted in an efficient one‐pot synthetic methodology for a disulfide‐containing biologically active peptide with over 50 amino acid residues. To the best of our knowledge, this is the first report detailing the development of highly water‐soluble Npys derivatives for use in peptide chemistry.

## 2 Results and Discussion

**Scheme 1:**
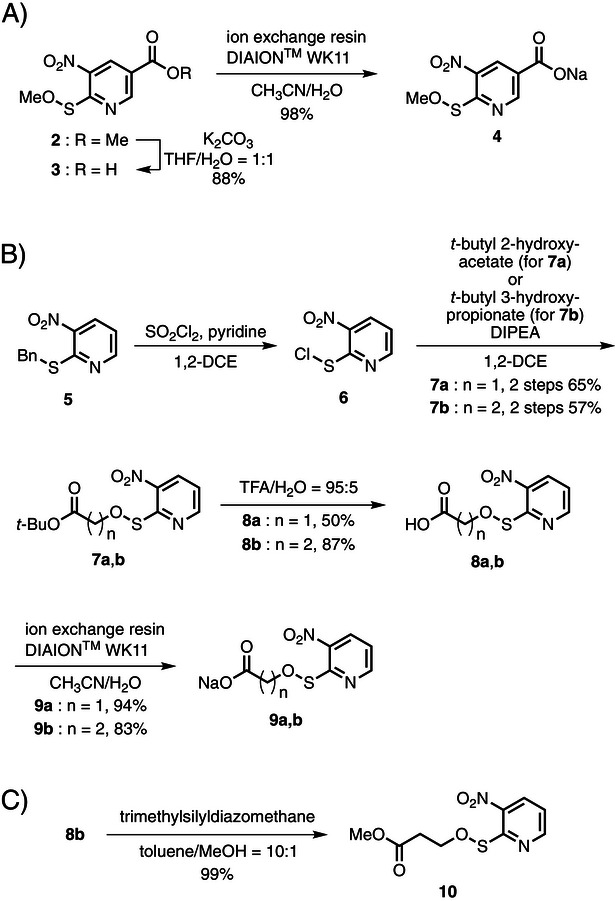
Synthesis of Npy‐sulfenate.

The synthesis of the Npy‐sulfenates is shown in Scheme 1. Initially, the methyl ester at C‐5 of the pyridine ring of Npys‐OMe^[^ ^24a^ ^]^ was hydrolyzed to the corresponding carboxylic acid **3** in 88% yield by treatment with K_2_CO_3_ in THF/H_2_O (1:1, *v/v*, pH 9–10) at room temperature (rt). Carboxylic acid **3** was converted into its monosodium salt by treating the CH_3_CN/H_2_O solution with a weakly acidic cation exchange resin (DIAION WK11).^[^ ^26^ ^]^ The desired sulfenate **4** was obtained in 98% yield after lyophilization (Scheme 1A). In Scheme 1B, commercially available 2‐(benzylthio)‐3‐nitropyridine (**5**, Npys‐Bn) was converted into Npys‐Cl (**6**) according to a reported synthetic procedure,^[^ ^27^ ^]^ and was immediately reacted with *t*‐butyl 2‐hydroxyacetate (for **7a**) or *t*‐butyl 3‐hydroxypropionate (for **7b**) in 1,2‐dichoroethane (1,2‐DCE) in the presence of *N*,*N*‐diisopropylethylamine (DIPEA). Sulfenates **7a** and **7b** were obtained in two steps from Npys‐Bn (**5**) in 65% and 57% yield, respectively, after workup and purification by silica gel column chromatography. *t*‐Butyl esters **7a** and **7b** were deprotected under acidic conditions, TFA/H_2_O (95/5, *v/v*) and the residue was purified by silica gel column chromatography to afford **8a** and **8b** in 50% and 87% yields, respectively. Finally, ion exchange led to the corresponding monosodium salts in good yields (**9a**: 94%; **9b**, Npys‐OPropNa: 83%). Carboxylic acid **8b** was treated with trimethyldiazomethane in toluene/MeOH (10:1, *v/v*) and the residue obtained after removing the solvent was purified by column chromatography to afford the corresponding methyl ester **10** in 99% yield.

The water solubilities of the Npy‐sulfenates were subsequently evaluated. Saturated solutions of the derivatives were prepared in distilled water, filtered through a centrifugal filter, and the filtrates analyzed by HPLC to determine their water solubilities,^[^ ^26^ ^]^ the results of which are shown in Figure 2A. Sulfenate **4** bearing the sodium carboxylate moiety at C‐5 of the pyridine ring exhibited a solubility greater than 50 mg/mL, which is over 350‐times higher than that of methyl ester **2** (0.13 mg/mL). Moreover, the water solubilities of sulfenates **9a** and **9b**, in which sodium carboxylates were introduced in place of the methyl sulfenate moieties, exhibited solubilities of 38 and >50 mg/mL, respectively. These values are over 64‐times higher than those of the original Npys‐OMe (**1**) (0.60 mg/mL). These results show that these sulfenates are highly water‐soluble owing to the ionic groups in their structures.

To examine whether or not the synthesized water‐soluble sulfenates function as oxidants for thiols of peptide, reduced oxytocin (**11**) was used. Peptide **11** was constructed using the standard Fmoc/*t*‐Bu SPPS protocol and Fmoc‐Rink amide SAL resin, and the resultant crude product was purified by preparative HPLC. A mixture of 1 equiv of each sulfenate relative to **11** in 1:3 (*v/v*) CH_3_CN/H_2_O (peptide concentration: 0.1 mM)^[^ ^24a^ ^]^ was stirred for 1 hour at rt and monitored over time by analytical HPLC, with the HPLC yield of oxytocin (**12**) at each time point calculated (Table S1 and Figures S2–S9). The HPLC yield of peptide **12** by sulfenates **1**, **2**, **4**, **9a**, and **9b** are shown on the left side of Figure 2B. Among them, Npys‐OPropNa (**9b**) efficiently oxidized **11** to cyclic disulfide peptide **12** in high yield (1 minute, 23%; 5 minutes, 68%; 10 minutes, 76%; 30 minutes, 82%; 60 minutes, 85%). Water‐soluble sulfenate **9a** gave peptide **12** in good yields (1 minute, 2%; 5 minutes, 13%; 10 minutes, 23%; 30 minutes, 45%; 60 minutes, 64%). In contrast, Npys‐OMe (**1**), the original compound devoid of a water‐solubilizing auxiliary group, gave a moderate yield of peptide **12** (40%), even after reaction for 1 hour, which shows that the introduction of the water‐solubilizing auxiliary group into the sulfenic acid methyl ester moiety of Npys‐OMe (**1**) led to improved yield of peptide **12**. On the other hand, sulfenate **4** afforded peptide **12** in 40% yield after 1 hour of reaction; this reactivity is similar to that of original derivative **2** (35%) devoid of the water‐solubilizing auxiliary group. The importance of the sodium carboxylate moiety to disulfide bond formation was investigated by oxidizing peptide **11** with a sulfenate in which the sodium carboxylate was replaced with a *t*‐butyl or methyl ester (**7b** or **10**) (Scheme 1). Sulfenates **7b** and **10** afforded oxytocin (**12**) in yields of 30% and 70%, respectively, which are lower than those obtained using Npys‐OPropNa (**9b**) (Figure 2B, right). These results indicate that the bulkiness of the ester moiety may hinder attack by the thiol of peptide at the sulfur atom of the sulfenate, leading to inefficient disulfide bond formation in peptide **11**. Sulfenate **8b** bearing the carboxylic acid moiety afforded oxytocin in 53% yield, which suggests that the sodium carboxylate moiety in Npys‐OPropNa (**9b**) is important for efficiently forming the disulfide bond in the peptide.

With Npys‐OPropNa (**9b**), which is both highly water‐soluble and efficiently forms disulfide bonds, in hand, we explored further synthetic applications of sulfenate **9b**. In particular, we explored the one‐pot synthesis of a cyclic disulfide peptide via NCL^[^ ^25^ ^]^ and subsequent sulfenate‐mediated disulfide bond formation. NCL facilitates the chemical syntheses of large peptides and proteins via chemoselective amide bond formation between C‐terminal thioester‐containing and N‐terminal Cys‐containing peptide fragments. NCL mainly involves the following two steps: i) thiol–thioester exchange between the C‐ and N‐terminal peptide fragments and ii) amide bond formation via intramolecular S‐to‐N acyl group transfer. Under typical NCL conditions, nucleophilic catalysis by a thiol additive, such as thiophenol or 4‐mercaptophenylacetic acid, is used to convert the alkyl thioester of the C‐terminal fragment to the corresponding more‐reactive aryl thioester in situ. A purification step is required to remove excess thiol additive from the NCL buffer before forming the disulfide bond in the linear peptide produced by NCL if the target peptide contains a disulfide bond. We focused on thiol‐additive‐free NCL^[^ ^25b^ ^]^ using a C‐terminal *N*‐methyl‐benzoimidazolinone‐containing (MeNbz‐containing)^[^ ^28^ ^]^ peptide fragment instead of the conventional NCL method. Sakamoto et al. reported that 1,2,4‐triazole facilitates NCL between the MeNbz‐containing peptide and a cysteinyl peptide in the absence of a thiol additive, and that thiol‐additive‐free NCL suppresses hydrolysis of the C‐terminal of the MeNbz‐containing peptide and improves NCL reactivity. In addition, the purification step otherwise needed to remove the thiol additive prior to peptide oxidation is avoided.^[^ ^25b^ ^]^

**Figure 3:**
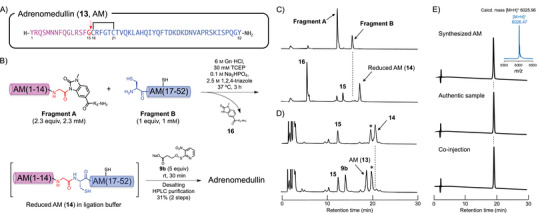
One‐pot synthesis of adrenomedullin (**13**, AM). A) Fragmenting the AM sequence. The red arrow between the Gly^15^ and Cys^16^ residues indicates the coupling site for thiol‐additive‐free native chemical ligation (NCL). B) Synthesis of AM, which comprises constructing reduced AM (**14**) by thiol‐additive‐free NCL and the direct sulfenate‐mediated disulfide bond formation involving Npys‐OPropNa (**9b**). C) HPLC traces acquired during the NCL. Upper: 0 hour. Lower: after 3 hours. HPLC conditions: linear 5% to 65% gradient of CH_3_CN in 0.1% aqueous TFA over 30 minutes at 1.0 mL/minute; detection: UV, *λ* = 230 nm. AM(1–15), **15**: H‐YRQSMNNFQGLRSFG‐OH. D) HPLC traces of disulfide bond formation in reduced AM (**14**). Upper: before reaction. Lower: after 30 minutes of oxidation. HPLC conditions: linear 15% to 45% gradient of CH_3_CN in 0.1% aqueous TFA over 30 minutes at 1.0 mL/minute; detection: UV, *λ* = 230 nm. *:4‐azidobenzoic acid. E) HPLC traces and MALDI‐TOF mass spectra of synthesized AM.

We selected adrenomedullin (**13**, AM), a hypotensive bioactive peptide with 52 amino acid residues, as the target in the one‐pot synthesis of a cyclic disulfide peptide via sulfenate‐mediated oxidation.^[^ ^29^ ^]^ We determined that the NCL ligation site lies between the Gly^15^ and Cys^16^ residues of the peptide and that there is no risk of epimerization (Figure 3A). The AM sequence was divided into two fragments: MeNbz‐containing N‐terminal fragment (**Fragment A**) and Cys‐containing C‐terminal fragment (**Fragment B**). **Fragment A** was prepared through several steps, including the introduction of penta‐Lys (K_5_) to enhance peptide solubility,^[^ ^30^ ^]^ coupling of 3‐[(Fmoc)amino]‐4‐(methylamino)benzoic acid to the K_5_ resin, and subsequent formation of a MeNbz structure on the peptidyl resin following a reported synthetic procedure.^[^ ^28^ ^]^ **Fragment B** was prepared using the conventional Fmoc/*t*‐Bu SPPS protocol (see Supporting Information). We planned to synthesize AM in a one‐pot process by constructing reduced AM (**14**) via thiol‐additive‐free NCL followed by direct Npy‐sulfenate‐mediated oxidation of the peptide (Figure 3B). Peptide **14** was first synthesized via thiol‐additive‐free NCL involving **Fragments A** and **B** in ligation buffer (6 M Gn·HCl, 30 mM tris(2‐carboxyethyl)phosphine (TCEP), 0.1 M Na_2_HPO_4_, 2.5 M 1,2,4‐triazole, pH 7)^[^ ^25b^ ^]^ (Table S2). The use of 1.1 equiv of **Fragment A** relative to **Fragment B** at rt afforded peptide **14** in 38% HPLC conversion in 3 hours. Extending the reaction time to 14 hours improved the HPLC conversion of the ligated product to 59%; however, **Fragment B** was not completely consumed. The ligation was considered inefficient due to the hydrolysis of **Fragment A** into the corresponding 15‐mer peptide (**15**, AM(1–15)) and the MeNbz‐containing linker (**16**, H‐MeNbz‐K_5_‐NH_2_) during the ligation process (Figure S12). We next investigated the number of equivalents of **Fragment A** and the reaction temperature in an effort to increase the yield of peptide **14**. Ligation using 2.3 equiv of **Fragment A** at rt for 3 hours led to an improved HPLC conversion of **14** (65%). Moreover, heating at 37 °C with same number of equivalents of **Fragment A** proceeded efficiently to afford linear peptide **14** in satisfactory HPLC yield conversion (94%) (Figure 3C).

We next examined sulfenate‐mediated disulfide bond formation in reduced AM (**14**) in ligation buffer. 4‐Azidobenzoic acid was added and the reaction mixture was stirred for 15 minutes at rt to quench excess TCEP^[^ ^25b^ ^]^ because the S─O bond of a sulfenate is well known to be easily cleaved by phosphine‐mediated reduction.^[^ ^24a^ ^]^ Peptide **14** was subsequently subjected to sulfenate‐mediated oxidation without purification or dilution of the reaction mixture. Figure 3D reveals that 5 equiv of Npys‐OPropNa (**9b**) led to the smooth formation of the disulfide bond between the Cys^16^ and Cys^21^ residues in linear peptide **14** within 30 minutes, resulting in an 89% conversion of AM. On the other hand, we confirmed that disulfide bond formation in peptide **14** does not proceed in the absence of Npys‐OPropNa (**9b**) (data not shown). The reaction mixture was desalted by gel filtration (PD‐10 column) following oxidation, and reverse‐phase HPLC afforded AM in 31% isolated yield based on **Fragment B**. MALDI‐TOF/MS revealed a molecular ion corresponding to AM (Figure 3E; *m/z* calcd. for C_264_H_407_N_80_O_77_S_3_ [M + H]^+^ 6025.96, found 6026.47), and HPLC revealed that the synthetic and authentic peptides exhibited consistent peaks when coinjected (Figure 3E). These results indicate that the one‐pot synthesis of AM involving the thiol‐additive‐free NCL of the MeNbz‐linker‐ and Cys‐containing fragments followed by sulfenate‐mediated disulfide bond formation was successful.

## 3 Conclusion

We developed novel Npy‐sulfenates by introducing sodium carboxylate moieties to improve water solubility. Among them, Npys‐OPropNa (**9b**) exhibited both higher water solubility and a superior ability to form disulfide bonds than original methyl sulfenate **1**. Moreover, Npys‐OPropNa (**9b**) can form the disulfide bond in reduced AM (**14**) prepared using thiol‐additive‐free NCL in aqueous buffer, providing the one‐pot synthetic methodology for preparing cyclic disulfide peptides. We expect that Npys‐OPropNa (**9b**), which acts as an efficient disulfide‐forming reagent in aqueous buffer, will contribute to the future disulfide bridging of biomolecules, including proteins and antibodies, which aggregate or denature in organic solvents, and is expected to be widely applied to oxidize proteins and antibodies.

## Conflict of Interests

The authors declare no conflict of interest.

## Supporting information



Supporting Information

## Data Availability

The data that support the findings of this study are available in the supplementary material of this article.
