# Supplementary material for: Development of Water‐Soluble 3‐Nitro‐2‐Pyridinesulfenate for Disulfide Bond Formation of Peptide Under Aqueous Conditions
Source: Chemistry. 2025 May 3;31(32):e202500855. doi: 10.1002/chem.202500855 (PMC12144866; doi:10.1002/chem.202500855)
Supplement: Supplementary file 1 — Supporting Information [file CHEM-31-e202500855-s001.pdf]

Supporting Information

Development of Water-soluble 3-Nitro-2-pyridinesulfenate for Disulfide Bond Formation of Peptide under Aqueous Conditions

Akihiro Taguchi*[a], Megumi Sakata[a], Ryusei Yamamoto[b], Hayate Shida[a], Saeka Kuraishi[a], Sho Konno[a], Kentaro Takayama[a,c], Atsuhiko Taniguchi[a], Yoshio Hayashi*[a][b]

[a]Department of Medicinal Chemistry, School of Pharmacy and [b]Laboratory of Medicinal Chemistry and Chemical Biology, School of Life Sciences Tokyo University of Pharmacy and Life Sciences, Hachioji, Tokyo 192-0392, Japan

[c]Laboratory of Environmental Biochemistry, Kyoto Pharmaceutical University, 5 Misasaginakauchi-cho, Yamashina, Kyoto 607-8414, Japan

*E-mail: ataguchi@toyaku.ac.jp, yhayashi@toyaku.ac.jp

Table of contents Page

1. General information S2
2. Synthetic method for disulfide compounds based on Npys-mediated disulfide bond formation S3
3. Synthesis of Npy-sulfenate S4
4. Evaluating the water-solubility of Npy-sufenate S8
5. Synthesis of reduced oxytocin S8
6. Disulfide bond formation in reduced oxytocin S8
7. Synthesis of adrenomedullin S18
8. ^{1}H and ^{13}C NMR spectra S23
9. References S32

1. General information

1-1. General synthetic method

^{1}H NMR spectra were acquired in $CDCl_3$, CD_3OD, DMSO-d_6, or D_2O on a Bruker AVANCE-III (400 MHz) spectrophotometer with TMS (0.00 ppm) or 3-(trimethylsilyl)propionic-2, 2, 3, 3-d_4 acid sodium salt (TSP-d_4, 0.00 ppm) as references. ^{13}C NMR spectra were acquired in $CDCl_3$, CD_3OD, DMSO-d_6, or D_2O on a Bruker AVANCE-III (100 MHz) spectrophotometer with $CDCl_3$ (77.05 ppm), CD_3OD (49.00 ppm), DMSO-d_6 (39.52 ppm), and TSP-d_4 (0.00 ppm) as references. The following abbreviations are used when reporting peak multiplicities: s, singlet; d, doublet; dd, double of doublets; t, triplet; and br s, broad singlet. Melting points were measured on a Yanaco MP-500D melting point apparatus. HRMS (TOF MS ES^+ or ES^-) was performed using Waters MICRO MASS LCT-premier and JEOL JMS-700 instruments. LRMS (ESI) was performed on a Shimadzu LCMS-2020 instrument. MALDI-TOF MS spectra were recorded on a Shimadzu Biotech AXIMA Assurance instrument using α-cyano-4-hydroxycinnamic acid as the matrix. Column chromatography was performed using silica gel 60N (spherical, neutral) (40–50 μm), and thin layer chromatography (TLC) was performed on precoated silica gel plates (0.25 mm, Merck Kieselgel $60F_{254}$). Compounds were visualized under UV light or by staining with phosphomolybdic acid or ninhydrin. Preparative HPLC was performed using a C18 reverse-phase column (19 mm × 150 mm; SunFire™ Prep C18 OBD™ 5 μm) and a binary solvent system. Analytical HPLC was performed using a C18 reverse-phase column (4.6 ID × 150 mm; Hitachi Lachrom IIC18(5 μm)) and a binary solvent system. Solvents and reagents were purchased from Kanto Chemical Co., Inc., Kokusan Chemical Co., Ltd., Mitsubishi Chemical Corporation, Peptide Institute, Inc., Tokyo Chemical Industry Co., Ltd., Wako Pure Chemical Industries, Ltd., and Watanabe Chemical Industries, Ltd.

1-2. Fmoc-solid phase peptide synthesis (Fmoc-SPPS) using an automated peptide synthesizer (Prelude®).

All reactions and washing steps were performed with bubbling N_2. Fmoc-Rink amide resin or Fmoc-Rink amide ChemMatrix® resin was placed in a reaction vessel. The resin was swelled with DMF (10 min × 3), after which 20% (v/v) piperidine/DMF solution was added to the resin and mixed by bubbling with N_2 (10 min × 2) in the reaction vessel. The resin was washed with DMF (30 s × 6) after removing the piperidine/DMF solution.

Fmoc-amino acid/DMF solution (0.2 M, 5.0 equiv), 1-hydroxy-7-azabenzotriazole (0.4 M, HOAt), 1-[bis-(dimethylamino)methylene]-1H-1,2,3-triazolo-[4,5-b]-pyridinium hexafluorophosphate-3-oxide (HATU)/DMF solution (5.0 equiv) and *N*,*N*-diisopropylethylamine (DIPEA)/DMF solution (0.8 M, 10 equiv) were added to the resin. The coupling reaction was performed by bubbling with N_2 for 30 min. The reaction mixture was filtered and the resulting resin was washed with DMF (30 s × 6). The desired peptidyl resins were prepared by repeating the Fmoc deprotection and coupling steps.

2. Synthetic method for disulfide compounds based on Npys-mediated disulfide bond formation

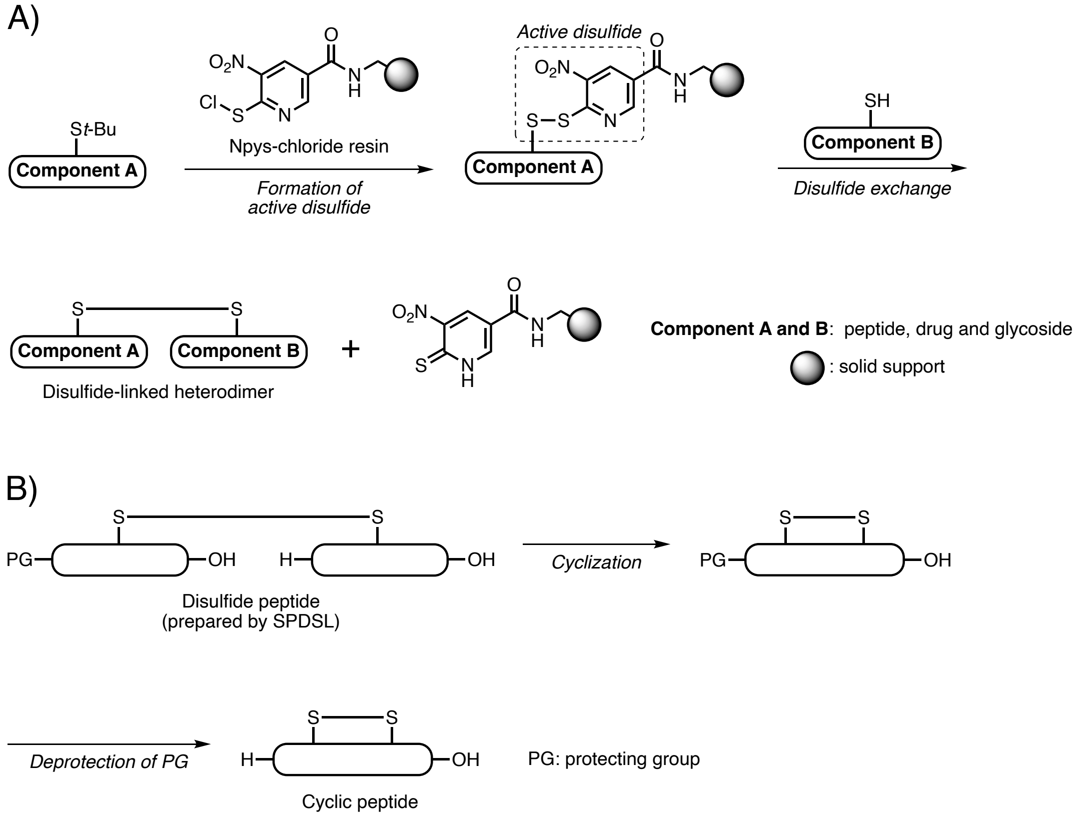

Scheme S1. One-pot solid-phase disulfide ligation (SPDSL)[1] (A) and disulfide-driven cyclic peptide synthesis (DdCPS)[2] (B).

3. Synthesis of Npy-sulfenate

Methyl 3-nitro-2-pyridinesulfenate (**1**, Npys-OMe) was synthesized according to a previously reported method.[3]

6-(Methoxythio)-5-nitronicotinic acid (3)

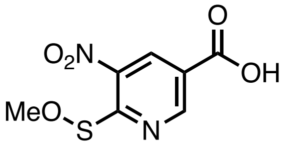

K_2CO_3 (31.2 mg, 0.226 mmol) was added to a solution of methyl 6-(benzylthio)-5-nitronicotinate[3] (**2**, 27.6 mg, 0.113 mmol) in THF/H_2O (1:1, 2 mL) at 0 ºC and stirred overnight at room temperature (rt), after which the solvent was removed under reduced pressure. The residue was washed with AcOEt, and the aqueous layer was acidified to pH 3 with 5% aqueous citric acid at 0 ºC and then extracted with AcOEt. The organic layer was washed with brine, dried over Na_2SO_4, filtered, and concentrated under reduced pressure to afford compound **3** (22.8 mg, 99.1 μmol, 88%) as a yellow solid; m.p 195.6-196.3 ºC; ^{1}H NMR (400 MHz, DMSO-d_6) δ 9.36 (d, J = 1.7 Hz, 1H), 8.80 (d, J = 1.7 Hz, 1H), 3.89 (s, 3H); ^{13}C NMR (100 MHz, DMSO-d_6) δ 167.2, 164.6, 154.5, 137.2, 134.0, 123.7, 65.2; HRMS (ESI) *m/z* calcd for $C_7H_7N_2O_5S$ $[M+H]^+$ 231.0076 found 231.0070.

Sodium 6-(methoxythio)-5-nitronicotinate (4)

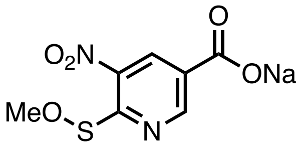

Compound **3** (51.8 mg, 0.225 mmol) was elated with a solvent of H_2O/CH_3CN (1:1) through ion exchange resin (DIAION® WK11) at rt.[4] The reaction mixture was filtrated with H_2O/CH_3CN (1:1) and concentrated. The residue was lyophilized to afford compound **4** (55.3 mg, 0.219 mmol, 98%) as a yellow solid; m.p 272.3 ºC (decomp.); ^{1}H NMR (400 MHz, DMSO-d_6) δ 9.27 (d, J = 1.6 Hz, 1H), 8.77 (d, J = 1.6 Hz, 1H), 3.86 (s, 3H); ^{13}C NMR (100 MHz, DMSO-d_6) δ 165.3, 163.1, 155.5, 137.0, 133.2, 132.4, 65.1; HRMS (ESI) *m/z* calcd for $C_7H_5N_2O_5S$ $[M-Na]^-$ 228.9919, found 228.9922.

tert-Butyl 2-(((3-nitropyridin-2-yl)thio)oxy)acetate (7a)

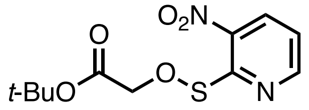

Sulfuryl chloride (144 μL, 1.79 mmol) and pyridine (32.8 μL, 0.406 mmol) were added to a solution of 2-(benzylthio)-3-nitropyridine **5** (200 mg, 0.812 mmol) in 1,2-dichloroethane (1,2-DCE, 5 mL) at rt. The mixture was stirred for 1 h at rt, after which the solvent was removed under reduced pressure. Hexane was added to the residue and removed under reduced pressure. The residue was used in the next step without further purification. *t*-Butyl glycolate (127 μL, 0.630 mmol) and DIPEA (559 μL, 3.25 mmol) were added a solution of the above residue in 1,2-DCE (3 mL) at 0 ºC and stirred for 2 h at rt. The mixture was diluted with $CHCl_3$ and the organic layer was washed with 10% aqueous citric acid, H_2O, and brine, dried over Na_2SO_4, filtered, and concentrated under reduced pressure. The residue was purified by silica gel flash column chromatography with hexane/AcOEt = 1:1 to afford compound **7a** (151 mg, 0.527 mmol, 65% over two steps) as a yellow oil. ^{1}H NMR (400 MHz, $CDCl_3$) δ 8.90 (dd, *J* = 1.6 and 4.5 Hz, 1H), 8.52 (dd, *J* = 1.6 and 8.3 Hz, 1H), 7.32 (dd, *J* = 4.5 and 8.3 Hz, 1H), 4.53 (s, 2H), 1.53 (s, 9H); ^{13}C NMR (100 MHz, $CDCl_3$) δ 168.1, 164.6, 154.6, 137.5, 133.2, 119.6, 82.6, 74.0, 28.2; HRMS (ESI) *m/z* calcd for $C_{11}H_{14}N_2O_5NaS$ $[M+Na]^+$ 309.0521, found 309.0518.

tert-Butyl 3-(((3-nitropyridin-2-yl)thio)oxy)propanoate (7b)

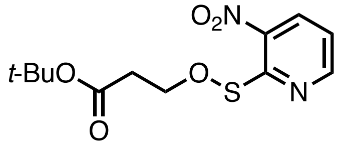

Compound **7b** was prepared in the same manner as compound **7a** using compound **5** (500 mg, 2.03 mmol) and *t*-butyl 3-hydroxypropionate (323 μL, 2.44 mmol). Compound **7b** (350 mg, 1.16 mmol, 57% over two steps) was obtained as a yellow solid; ^{1}H NMR (400 MHz, $CDCl_3$) δ 8.90 (dd, *J* = 1.6 and 4.6 Hz, 1H), 8.51 (dd, *J* = 1.6 and 8.3 Hz, 1H), 7.30 (dd, *J* = 4.5 and 8.3 Hz, 1H), 4.32 (t, *J* = 6.6 Hz, 2H), 2.75 (t, *J* = 6.5 Hz, 2H), 1.48 (s, 9H); ^{13}C NMR (100 MHz, $CDCl_3$) δ 170.0, 165.1, 154.6, 137.5, 133.2, 119.4, 81.1, 74.10, 36.7, 28.1; HRMS (ESI) *m/z* calcd for $C_{12}H_{16}N_2O_5NaS$ $[M+Na]^+$ 323.0678, found 323.0678.

2-(((3-Nitropyridin-2-yl)thio)oxy)acetic acid (8a)

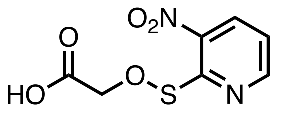

TFA/H_2O (95:5, v/v, 7 mL) was added to compound **7a** (151 mg, 0.527 mmol) at rt and stirred for 2 h. The solvent was removed under reduced pressure and the residue was purified by silica gel flash column chromatography with $CHCl_3$/MeOH = 50:1 to afford compound **8a** (60.6 mg, 0.263 mmol, 50%) as a yellow solid; m.p 146.4-147.2 ºC; ^{1}H NMR (400 MHz, $CDCl_3$) δ 13.96 (br s, 1H), 8.92 (dd, *J* = 1.6 and 4.8 Hz, 1H), 8.68 (dd, *J* = 1.6 and 8.3 Hz, 1H), 7.52 (dd, *J* = 4.8 and 8.3 Hz, 1H), 4.65 (s, 2H); ^{13}C NMR (100 MHz, CD_3OD) δ 172.6, 164.7, 155.8, 139.1, 134.8, 121.5, 74.7; HRMS (ESI) *m/z* calcd for $C_7H_6N_2O_5NaS$ $[M+Na]^+$ 252.9895, found 252.9893.

3-(((3-Nitropyridin-2-yl)thio)oxy)propanoic acid (8b)

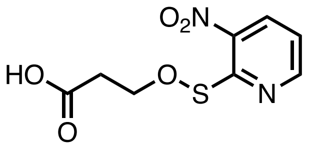

Compound **8b** was prepared in the same manner as compound **8a** using compound **7b** (181 mg, 0.603 mmol). The residue was purified by silica gel flash column chromatography with $CHCl_3$/MeOH = 50:1 to afford compound **8b**. Compound **8b** (128 mg, 0.524 mmol, 87%) was obtained as a yellow solid; m.p 110.8-112.3 ºC; ^{1}H NMR (400 MHz, $CDCl_3$) δ 8.90 (dd, *J* = 1.6 and 4.7 Hz, 1H), 8.58 (dd, *J* = 1.6 and 8.3 Hz, 1H), 7.38 (dd, *J* = 4.7 and 8.3 Hz, 1H), 4.30 (t, *J* = 5.9 Hz, 2H), 2.90 (t, *J* = 5.8 Hz, 2H); ^{13}C NMR (100 MHz, $CDCl_3$) δ 174.0, 164.3, 154.3, 137.7, 133.9, 120.0, 72.7, 35.8; HRMS (ESI) *m/z* calcd for $C_8H_8N_2O_5NaS$ $[M+Na]^+$ 267.0052, found 267.0051.

Sodium 2-(((3-nitropyridin-2-yl)thio)oxy)acetate (9a)

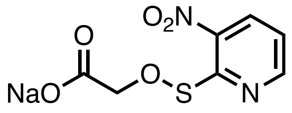

Compound **9a** was prepared in the same manner as compound **4** using compound **8a** (100 mg, 0.434 mmol). Compound **9a** (103 mg, 0.408 mmol, 94%) was obtained as a yellow solid; m.p 179.1 ºC (decomp.); ^{1}H NMR (400 MHz, CD_3OD) δ 8.90 (dd, *J* = 1.6 and 4.6

Hz, 1H), 8.62 (dd, J = 1.6 and 8.3 Hz, 1H), 7.44 (dd, J = 4.6 and 8.3 Hz, 1H), 4.42 (s, 2H); ^{13}C NMR (100 MHz, CD_3OD) δ 175.7, 165.5, 155.8, 139.1, 134.7, 121.2, 76.8; HRMS (ESI) *m/z* calcd for $C_7H_6N_2O_5NaS$ $[M+H]^+$ 252.9895, found 252.9876.

Sodium 3-(((3-nitropyridin-2-yl)thio)oxy)propanoate (Npys-OPropNa, 9b)

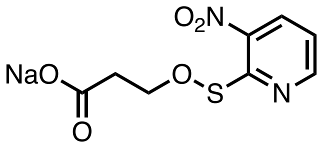

Compound **9b** was prepared in the same manner as compound **4** using compound **8b** (11.7 mg, 0.048 mmol). Compound **9b** (10.57 mg, 0.040 mmol, 83%) was obtained as a yellow solid; m.p 153.3 ºC (decomp.); ^{1}H NMR (400 MHz, DMSO-d_6) δ 8.98 (dd, J = 1.6 and 4.5 Hz, 1H), 8.63 (dd, J = 1.6 and 8.4 Hz, 1H), 7.50 (dd, J = 4.5 and 8.4 Hz, 1H), 4.16 (t, J = 7.2 Hz, 2H), 2.41 (t, J = 7.2 Hz, 2H); ^{13}C NMR (100 MHz, D_2O) δ 182.5, 166.2, 157.7, 140.8, 137.2, 123.5, 78.4, 41.1; HRMS (ESI) *m/z* calcd for $C_8H_8N_2O_5NaS$ $[M+H]^+$ 267.0052, found 267.0033.

Methyl 3-(((3-nitropyridin-2-yl)thio)oxy)propanoate (10)

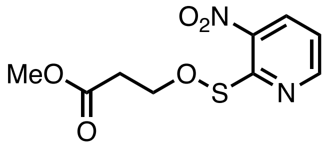

Trimethylsilyldiazomethane (10% in hexane, 1.36 mL, 0.819 mmol) was added dropwise to a solution of compound **8b** (100 mg, 0.409 mmol) in toluene/MeOH (10:1, 4 mL) at 0 ºC and stirred at rt for 30 min. The solvent was removed under reduced pressure and the residue was purified by silica gel flash column chromatography with hexane/AcOEt = 3:1 to afford compound **10** (105 mg, 0.407 mmol, 99%) as a yellow solid; ^{1}H NMR (400 MHz, CD_3OD) δ 8.90 (dd, J = 1.6 and 4.6 Hz, 1H), 8.61 (dd, J = 1.5 and 8.4 Hz, 1H), 7.44 (dd, J = 4.6 and 8.3 Hz, 1H), 4.29 (t, J = 6.2 Hz, 2H), 3.72 (s, 3H), 2.80 (t, J = 6.2 Hz, 2H). ^{13}C NMR (100 MHz, CD_3OD) δ 173.0, 165.2, 155.8, 138.9, 134.6, 121.2, 74.8, 52.3, 36.1; HRMS (ESI) *m/z* calcd for $C_9H_{11}N_2O_5S$ $[M+H]^+$ 259.0389, found 259.0389.

4. Evaluating the water-solubility of Npy-sulfenate

The water solubility of each Npy-sulfenate was evaluated according to a previously reported procedure.[4] The Npy-sulfenate was dissolved in distilled water to saturation and shaken vigorously. The saturated solution was filtered through a centrifugal filter (0.45-nm filter unit, Ultrafree®–MC, Millipore) and the filtrate was subjected to HPLC to determine water solubility.

5. Synthesis of reduced oxytocin

Reduced oxytocin (**11**) was prepared according to a previously reported procedure.[3] Fmoc-Rink amide resin (40 μmol, loading rate: 0.58 mmol/g, 69.5 mg) was subjected to the automated peptide synthesis protocol described above. After peptide elongation, the peptidyl resin was deprotected with a TFA cocktail (TFA/H_2O/1,2-ethanedithiol (EDT)/triisopropylsilane (TIPS) = 95:2.5:2.5:1, v/v/v/v, 4 mL), and the mixture was stirred for 3 h at rt, filtered, and the TFA removed under a stream of N_2. The residue was precipitated from ice-chilled Et_2O, washed twice with ice-chilled Et_2O, and dried *in vacuo*. The crude product was purified by reversed-phase HPLC to give peptide **11** as a white powder (15.0 mg, 13.4 μmol, 33%).

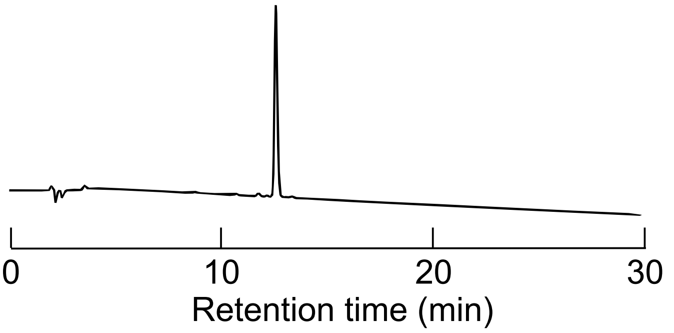

Figure S1. HPLC chart of reduced oxytocin (**11**). HPLC conditions: linear 15% to 45% gradient of CH_3CN in 0.1% aqueous TFA over 30 min at 1.0 mL/min, with detection at 230 nm.

6. Disulfide bond formation in reduced oxytocin

Npy-sulfenate-mediated disulfide bond formation in peptide **11** was performed using a previously reported procedure with slight modifications.[3]

A solution of 0.1 mM reduced oxytocin in H_2O/CH_3CN (3:1, v/v) was prepared, and an aliquot of this peptide solution (prior to the addition of Npy-sulfenate) was subjected to

HPLC. Npy-sulfenate (1.0 equiv) was added to a solution of peptide **11** (1.0 equiv) and the reaction mixture was stirred at rt. An aliquot of the reaction mixture was subjected to HPLC after 1 min, 5 min, 10 min, 30 min, and 1 h.

Table S1. Disulfide bond formation in reduced oxytocin using Npy-sulfenate

Entry	Compd.	HPLC yield of oxytocin (%)[a]				
		1 min	5 min	10 min	30 min	60 min
1	**1**	0.8	5	11	24	40
2	**2**	1	4	8	21	35
3	**4**	1	5	11	25	40
4	**7b**	7	11	17	17	30
5	**8b**	11	28	30	45	53
6	**9a**	2	13	23	45	64
7	**9b**	23	68	76	82	85
8	**10**	16	29	43	63	70

[a] HPLC yield (%) of oxytocin (**12**) in reaction mixture. The yield was calculated by using a calibration curve of **12**.

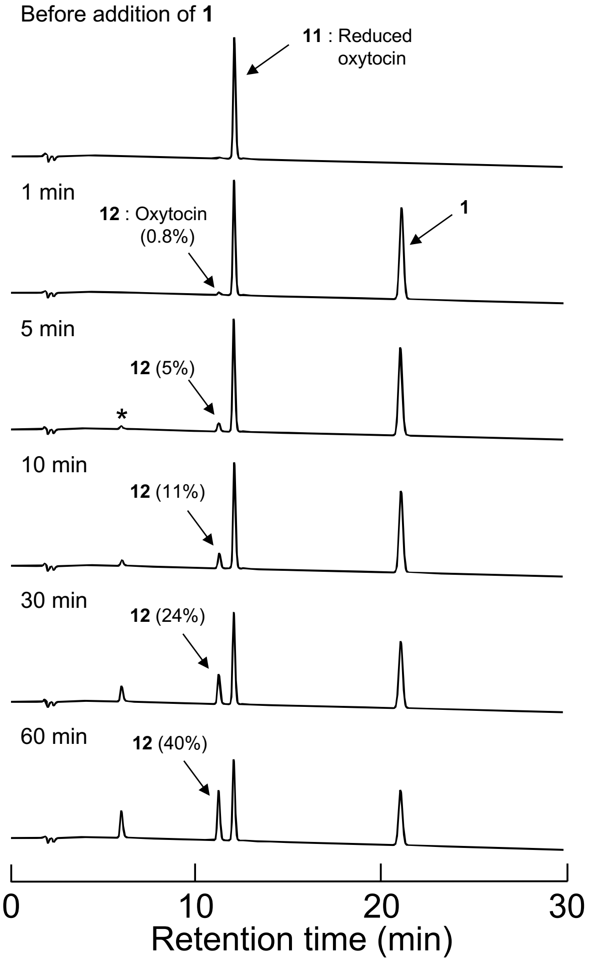

Figure S2. HPLC traces of time course analysis (Entry 1 in Table S1). HPLC conditions: linear 15% to 45% gradient of CH_3CN in 0.1% aqueous TFA over 30 min at a flow rate of 1.0 mL/min, with detection at 230 nm. *Non-peptide peak.

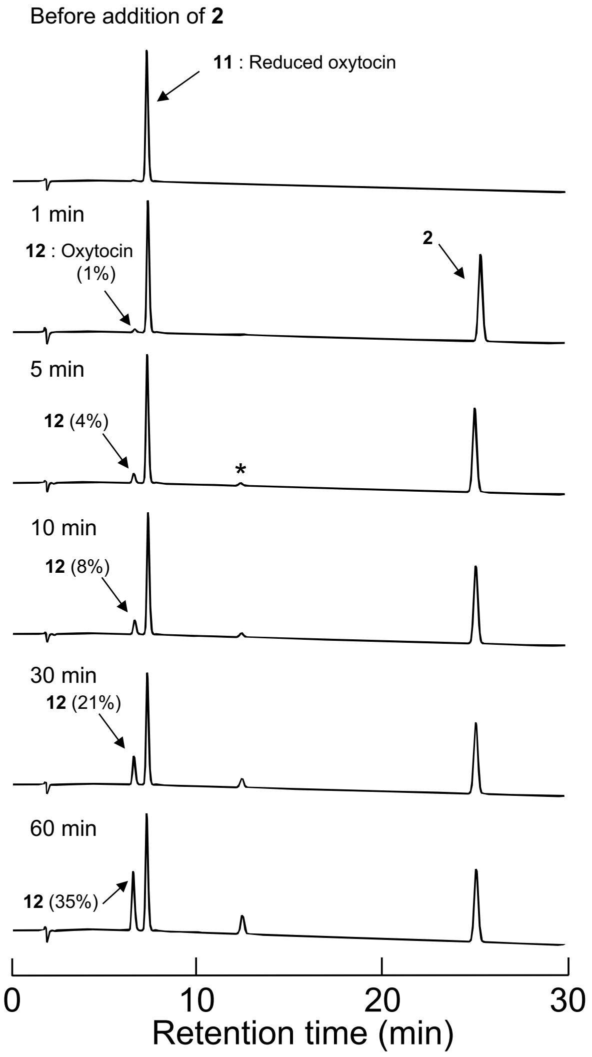

Figure S3. HPLC traces of time course analysis (Entry 2 in Table S1). HPLC conditions: linear 20% to 50% gradient of CH_3CN in 0.1% aqueous TFA over 30 min at a flow rate of 1.0 mL/min, with detection at 230 nm. *Non-peptide peak.

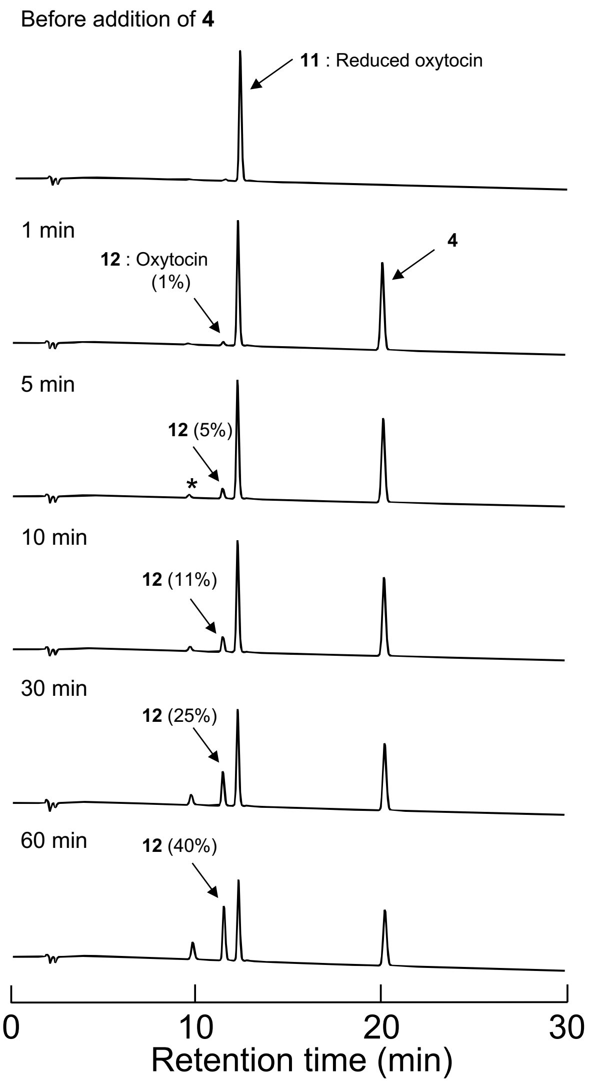

Figure S4. HPLC traces of time course analysis (Entry 3 in Table S1). HPLC conditions: linear 15% to 45% gradient of CH_3CN in 0.1% aqueous TFA over 30 min at a flow rate of 1.0 mL/min, with detection at 230 nm. *Non-peptide peak.

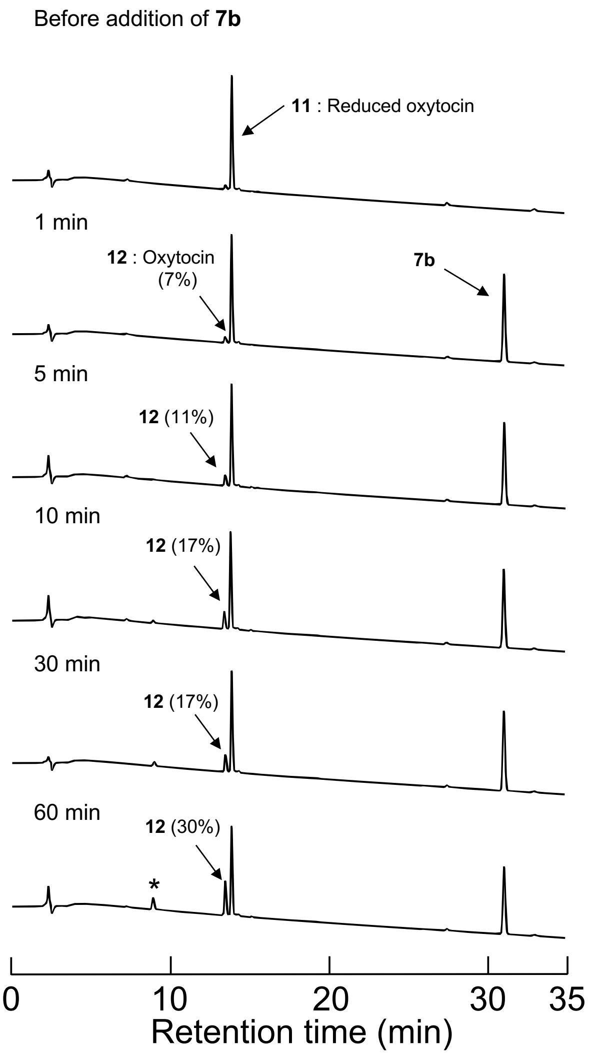

Figure S5. HPLC traces of time course analysis (Entry 4 in Table S1). HPLC conditions: linear 5% to 75% gradient of CH_3CN in 0.1% aqueous TFA over 35 min at a flow rate of 1.0 mL/min, with detection at 230 nm. *Non-peptide peak.

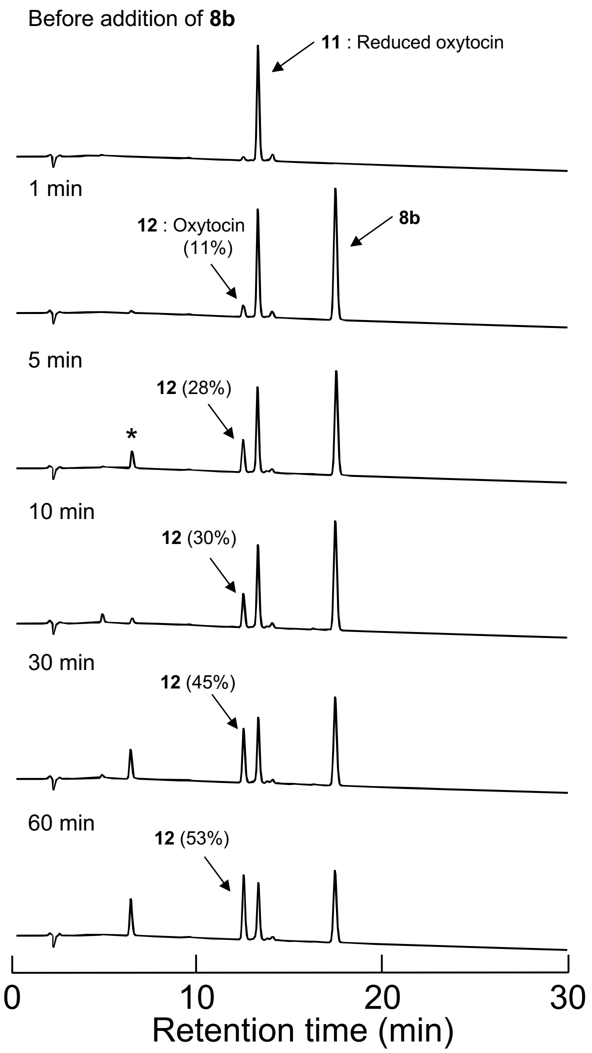

Figure S6. HPLC traces of time course analysis (Entry 5 in Table S1). HPLC conditions: linear 15% to 45% gradient of CH_3CN in 0.1% aqueous TFA over 30 min at a flow rate of 1.0 mL/min, with detection at 230 nm. *Non-peptide peak.

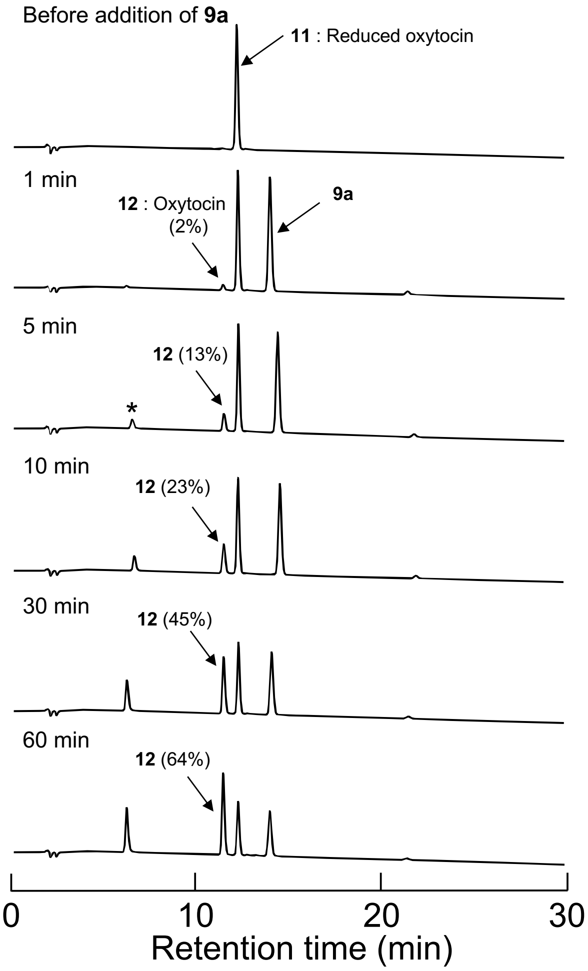

Figure S7. HPLC traces of time course analysis (Entry 6 in Table S1). HPLC conditions: linear 15% to 45% gradient of CH_3CN in 0.1% aqueous TFA over 30 min at a flow rate of 1.0 mL/min, with detection at 230 nm. *Non-peptide peak.

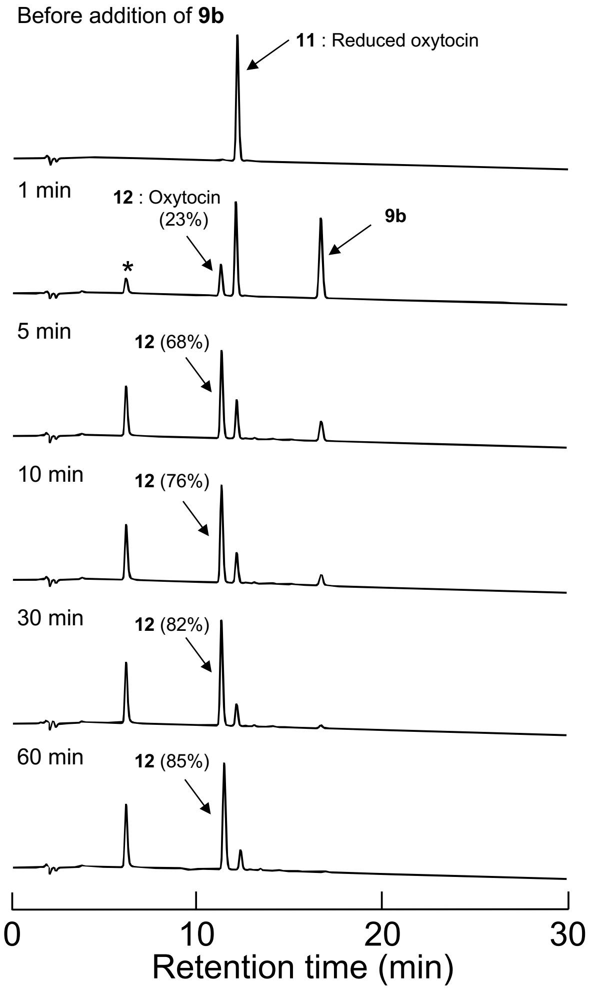

Figure S8. HPLC traces of time course analysis (Entry 7 in Table S1). HPLC conditions: linear 15% to 45% gradient of CH_3CN in 0.1% aqueous TFA over 30 min at a flow rate of 1.0 mL/min, with detection at 230 nm. *Non-peptide peak.

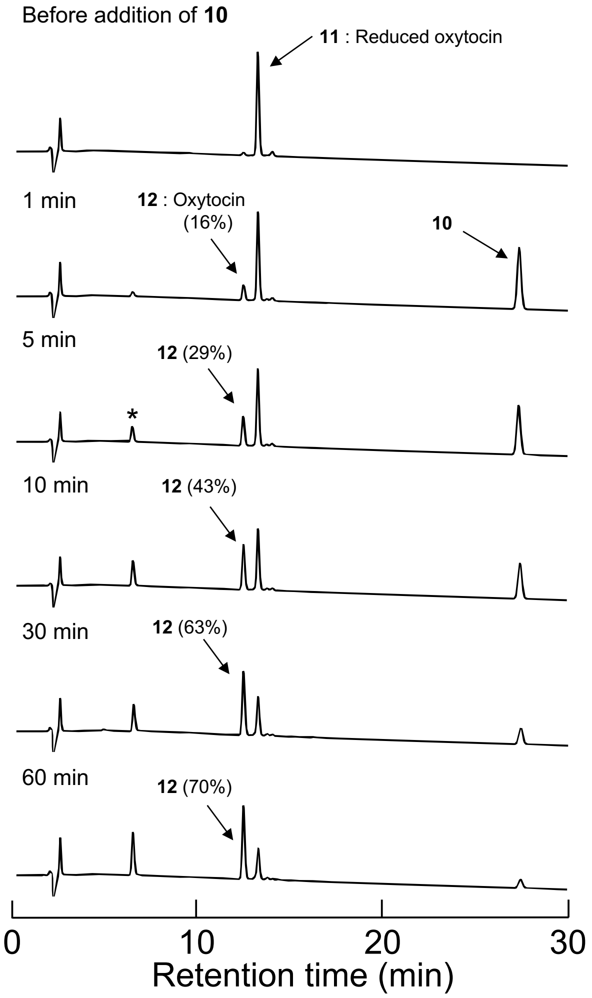

Figure S9. HPLC traces of time course analysis (Entry 8 in Table S1). HPLC conditions: linear 15% to 45% gradient of CH_3CN in 0.1% aqueous TFA over 30 min at a flow rate of 1.0 mL/min, with detection at 230 nm. *Non-peptide peak.

7. Synthesis of adrenomedullin

7-1. Synthesis of **Fragment A**

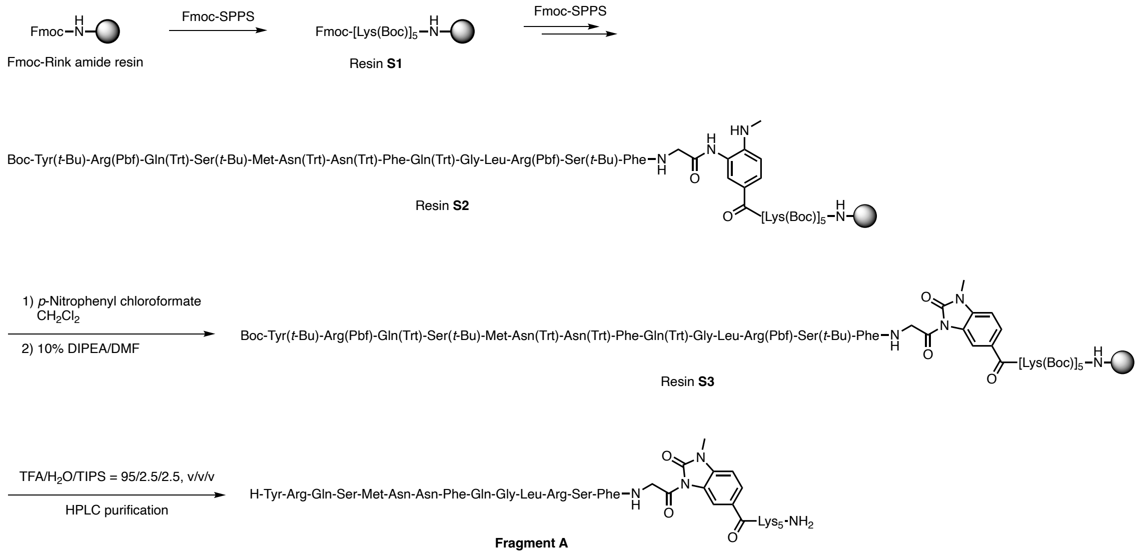

Fmoc-[Lys(Boc)]$_5$-NH-resin (resin **S1**) was prepared by subjecting Fmoc-Rink amide resin (255 mg, 120 μmol) to the Fmoc-based SPPS method according to the Prelude® automatic protocol, after which [(Fmoc)amino]-4-(methylamino)benzoic acid and Fmoc-Gly-OH were manually coupled to resin **S1** according to a previously described procedure.[5] Resin **S1** was subjected to the automated peptide synthesis protocol to prepare resin **S2**. *p*-Nitrophenyl chloroformate was added to resin **S2**, and the resultant resin was treated with a 10% DIPEA/DMF solution to form the MeNbz structure[5] (resin **S3**). Resin **S3** was treated with a TFA cocktail (TFA/H_2O/TIS = 95:2.5:2.5, v/v/v, 6 mL) and the mixture was stirred for 3 h at rt. After filtration, TFA was removed under a stream of N_2 and the residue was precipitated from ice-chilled ether, washed twice with ice-chilled ether, and dried *in vacuo*. The crude product was purified by reversed-phase HPLC to afford **Fragment A** (52.1 mg, 14.8 μmol, 12%). HRMS (ESI) *m/z* calcd for $C_{117}H_{185}N_{38}O_{29}S$ [M+H]$^+$ 2618.3890, found 2618.3882.

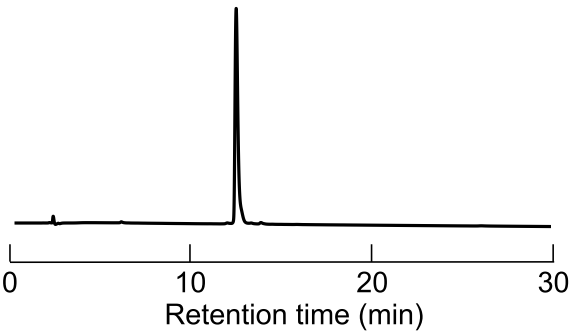

Figure S10. HPLC chart of **Fragment A**. HPLC conditions: linear 5% to 65% gradient of CH_3CN in 0.1% aqueous TFA over 30 min at a flow rate of 1.0 mL/min, with detection at 230 nm.

7-2. Synthesis of **Fragment B**

Fmoc-Rink amide ChemMatrix® resin (0.41 mmol/g, 293 mg, 120 μmol) was subjected to the Fmoc-based SPPS method according to the Prelude® automatic protocol to afford H-Cys(Trt)-Arg(Pbf)-Phe-Gly-Thr(*t*-Bu)-Cys(Trt)-Thr(*t*-Bu)-Val-Gln(Trt)-Lys(Boc)-Leu-Ala-His(Trt)-Gln(Trt)-Ile-Tyr(*t*-Bu)-Gln(Trt)-Phe-Thr(*t*-Bu)-Asp(O*t*-Bu)-Lys(Boc)-Asp(O*t*-Bu)-Lys(Boc)-Asp(O*t*-Bu)-Asn(Trt)-Val-Ala-Pro-Arg(Pbf)-Ser(*t*-Bu)-Lys(Boc)-Ile-Ser(*t*-Bu)-Pro-Gln(Trt)-Gly-Tyr(*t*-Bu)-NH-resin (473 mg). The resin was treated with a TFA cocktail (TFA/H_2O/TIS/EDT = 94:2.5:1.0:2.5, v/v/v/v, 15 mL) and the mixture was stirred for 3 h at rt. After filtration, TFA was removed under a stream of N_2 and the residue was precipitated from ice-chilled ether, washed twice with ice-chilled ether, and dried *in vacuo*. The crude product was purified by reversed-phase HPLC to afford **Fragment B** (205 mg, 40.6 mmol, 34%). HRMS (ESI) *m/z* calcd for $C_{186}H_{294}N_{55}O_{55}S_2$ $[M+H]^+$ 4242.1342, found 4242.1396.

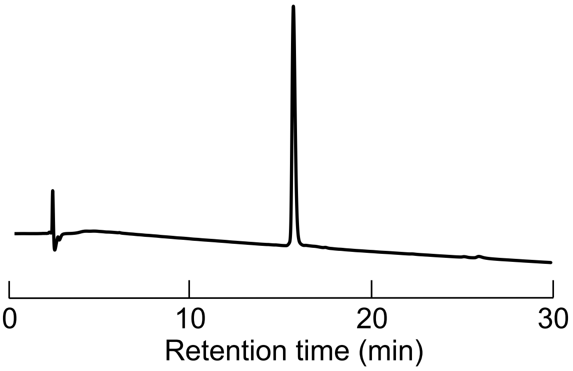

Figure S11. HPLC chart of **Fragment B**. HPLC conditions: linear 5% to 65% gradient of CH_3CN in 0.1% aqueous TFA over 30 min at a flow rate of 1.0 mL/min, with detection at 230 nm.

7-3. Native chemical ligation and Npy-sulfenate-mediated disulfide bond formation

Native chemical ligation was performed according to a known synthetic procedure.[6]

Fragment A (1.1, 2.0, or 2.3 equiv) and **Fragment B** (1.0 equiv) were dissolved in NCL buffer (1.0 mL, 6 M Gn·HCl, 100 mM Na_2HPO_4, 2.5 M 1,2,4-triazole, 30 mM TCEP, pH 7), and the mixture was stirred for 3 h at rt or 37 ºC. The ligation product (reduced AM (**14**)) in the reaction mixture was analyzed using HPLC with a linear 5% to 65% gradient of CH_3CN in 0.1% aqueous TFA over 30 min at 1.0 mL/min, with detection at 230 nm. The HPLC conversions of peptide **14** are shown in Table S2 with the corresponding HPLC traces displayed in Figures S12–S14.

Reduced AM (**14**): MALDI-TOF/MS *m/z* calcd for $C_{264}H_{409}N_{80}O_{77}S_3$ $[M+H]^+$ 6027.97, found 6028.99.

Hydrolyzed peptide (**15**, AM(1-15), H-YRQSMNNFQGLRSFG-OH): LRMS *m/z* calcd for $C_{78}H_{119}N_{25}O_{23}S^{2+}$ $[M+2H]^{2+}$ 902.93, found 903.50.

MeNbz-containing linker (**16**, H-MeNbz-K5-NH2): LRMS *m/z* calcd for $C_{39}H_{71}N_{13}O_7{}^{2+}$ $[M+2H]^{2+}$ 416.78, found 417.00.

Table S2. Thiol-additive-free NCL between **Fragments A** and **B**

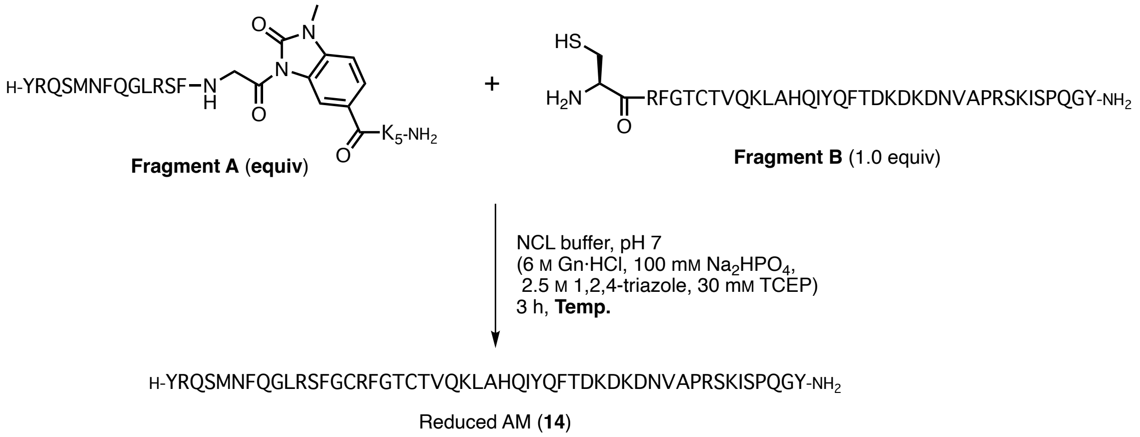

Entry	**Fragment A** (equiv)	Temp.	Conversion of **14** (%)[a]
1	1.1	rt	38 (59)[b]
2	2.3	rt	65
3	2.3	37 ºC	94

[a] Conversion (%) of reduced AM (**14**) in reaction mixture after 3 h. The value was calculated using the following formula: [(peak area of peptide **14**)/(sum of peak area of peptide **14** and **Fragment B**)] x 100.
[b] Conversion (%) of reduced AM (**14**) after 14 h.

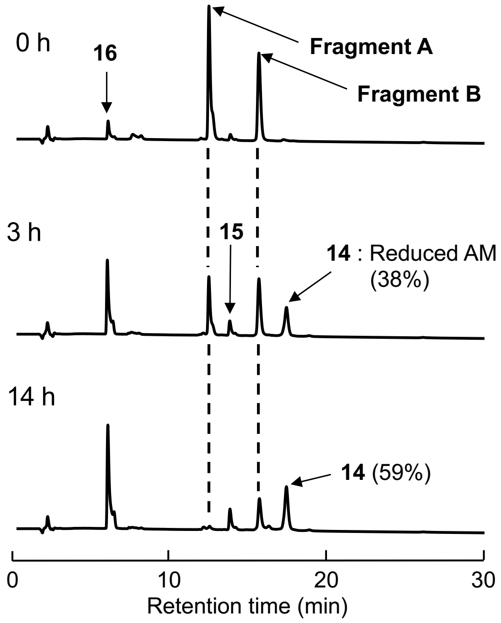

Figure S12. HPLC traces of Entry 1 in Table S2.

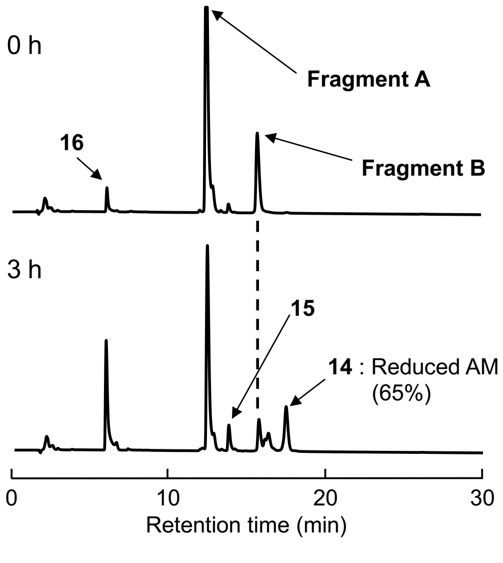

Figure S13. HPLC traces of Entry 2 in Table S2.

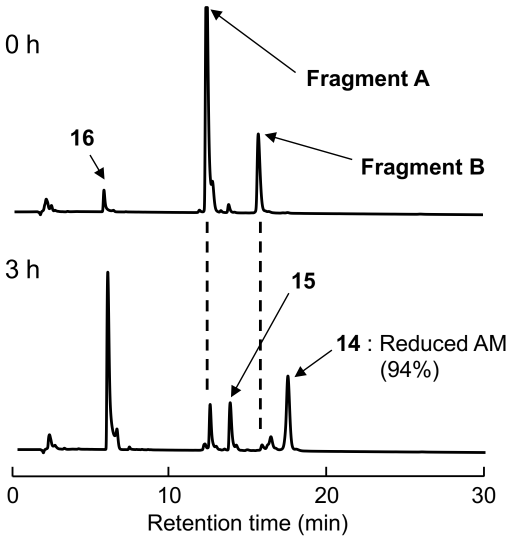

Figure S14. HPLC traces of Entry 3 in Table S2.

7-4. Npy-sulfenate-mediated disulfide bond formation in peptide **14**

A reaction mixture containing reduced AM (**14**) was prepared from **Fragment A** (2.5 mg, 0.69 μmol) and **Fragment B** (1.5 mg, 0.30 μmol) according to the conditions of Entry 3 in Table S2 (HPLC traces are shown in Figure 3C in the main text.). 4-Azidobenzoic acid (2.0 mg, 12 μmol) was added to the reaction mixture after which it was stirred for 15 min at rt.[6] Npys-OPropNa (**9b**) in H_2O (0.10 M, 15 μL, 1.5 μmol) was then added to above reaction mixture at rt. The HPLC traces for this reaction are shown in Figure 3D in the main text (HPLC conditions: linear 15% to 45% gradient of CH_3CN in 0.1% aqueous TFA over 30 min at a flow rate of 1.0 mL/min, with detection at 230 nm). Conversion of AM was 89%. The value was calculated using the following formula: [(peak area of peptide **13**)/(sum of peak area of peptides **13** and **14**)] x 100. After stirring for 30 min at rt, the reaction mixture was passed through a PD-10 desalting column. The crude product was purified by preparative HPLC using 0.1% aqueous TFA-CH_3CN to afford adrenomedullin (0.73 mg, 0.31 mmol, 31% based on **Fragment B**) as a white solid. MALDI-TOF/MS *m/z* calcd for $C_{264}H_{407}N_{80}O_{77}S_3$ $[M+H]^+$ 6025.96, found 6026.47. The HPLC traces of synthetic and authentic peptides are shown in Figure 3E in the main text (HPLC conditions: linear 15% to 45% gradient of CH_3CN in 0.1% aqueous TFA over 30 min at a flow rate of 1.0 mL/min, with detection at 230 nm). Human adrenomedullin for authentic sample was purchased from Peptide Institute, Inc.

^{1}H NMR (400 MHz, DMSO-*d*6)

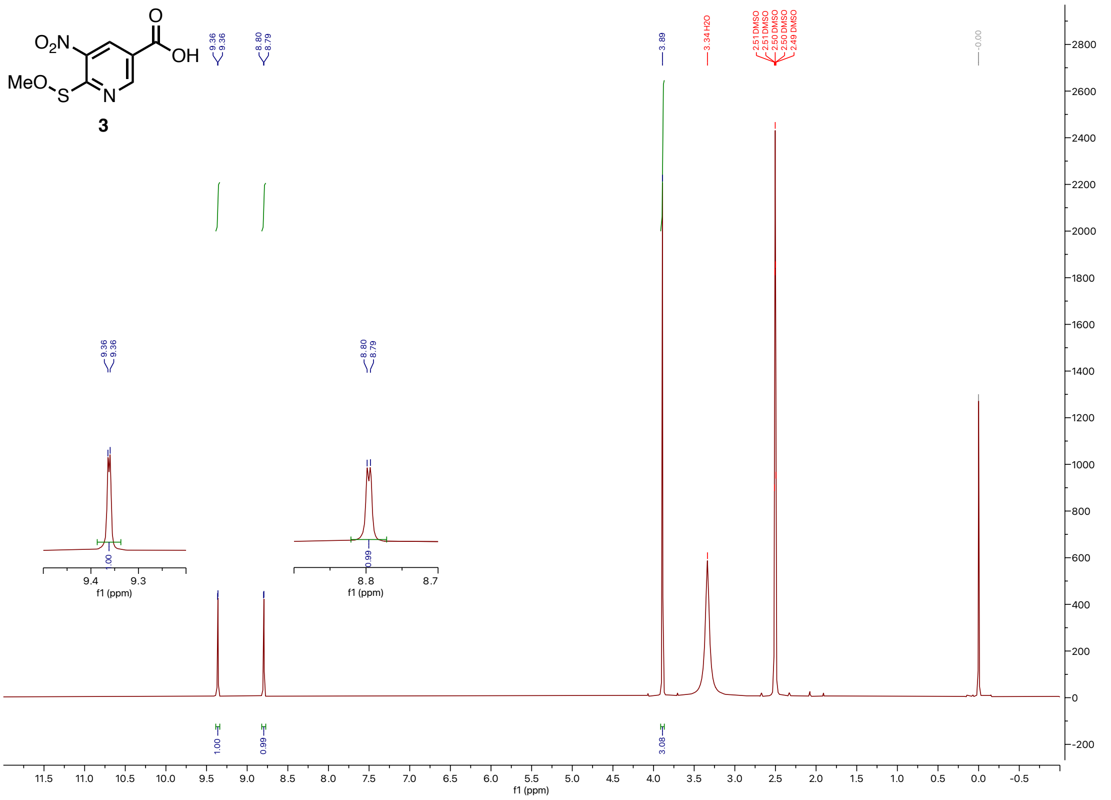

^{13}C NMR (100 MHz, DMSO-*d*6)

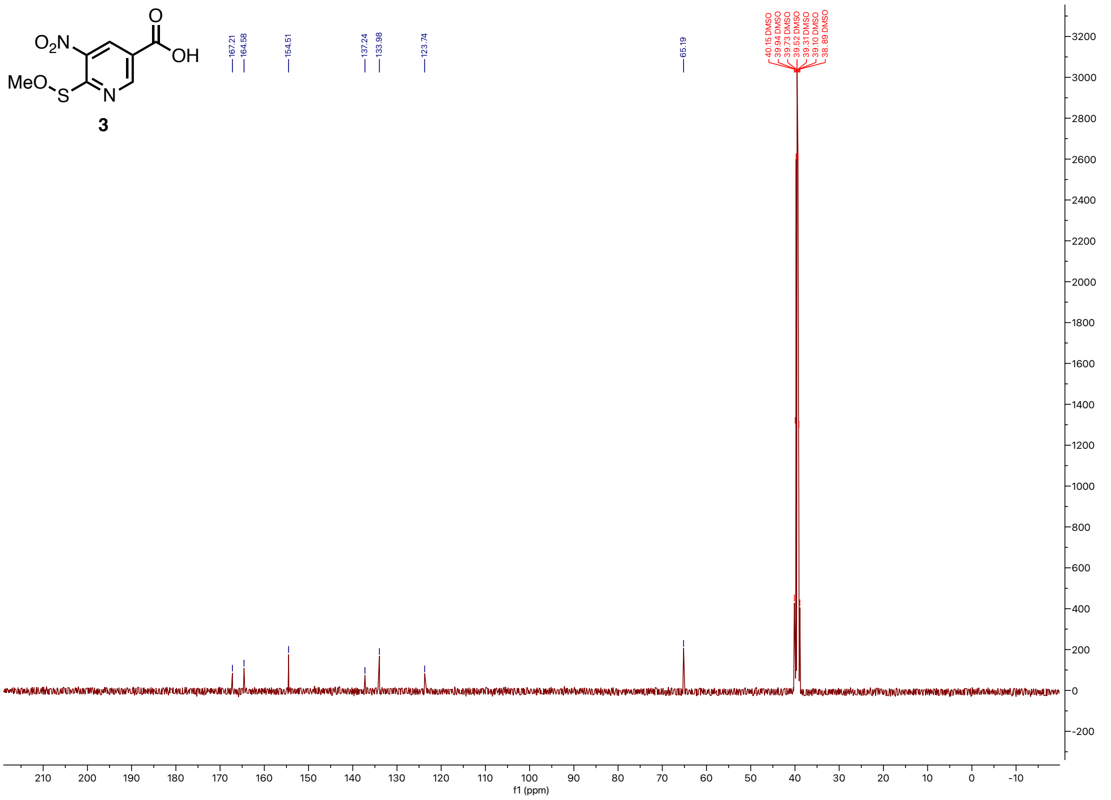

^{1}H NMR (400 MHz, DMSO-*d*6)

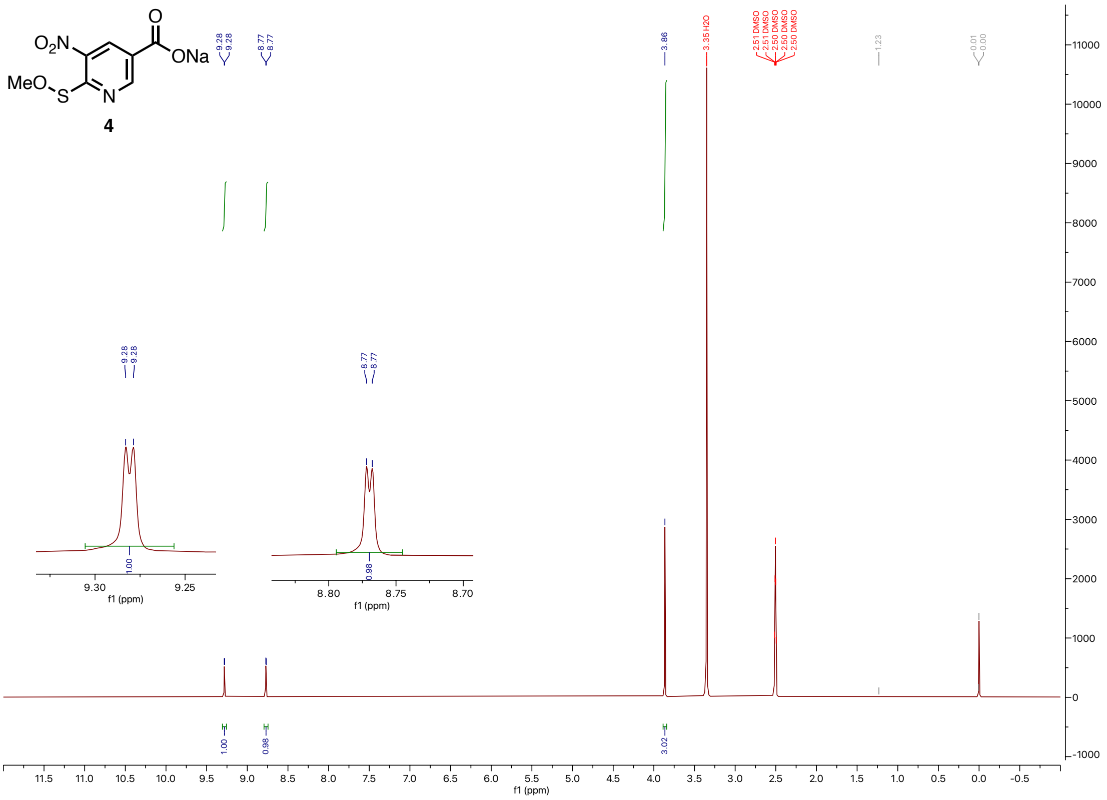

^{13}C NMR (100 MHz, DMSO-*d*6)

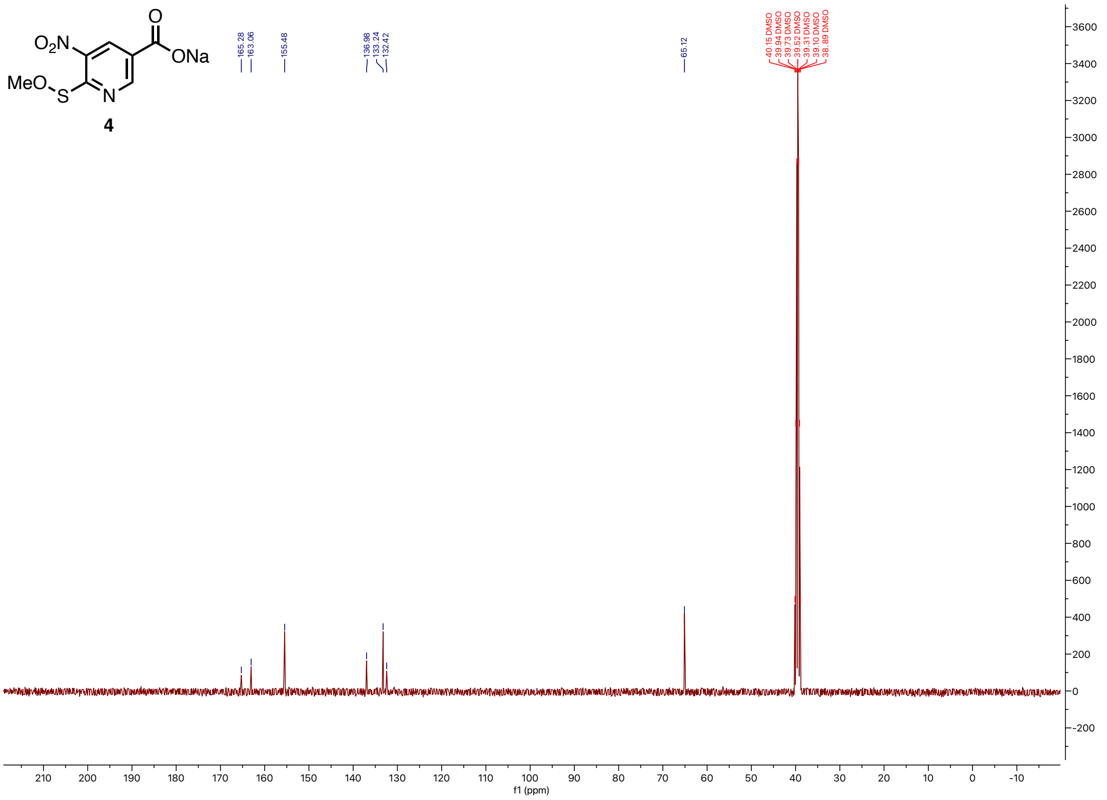

^{1}H NMR (400 MHz, $CDCl_3$)

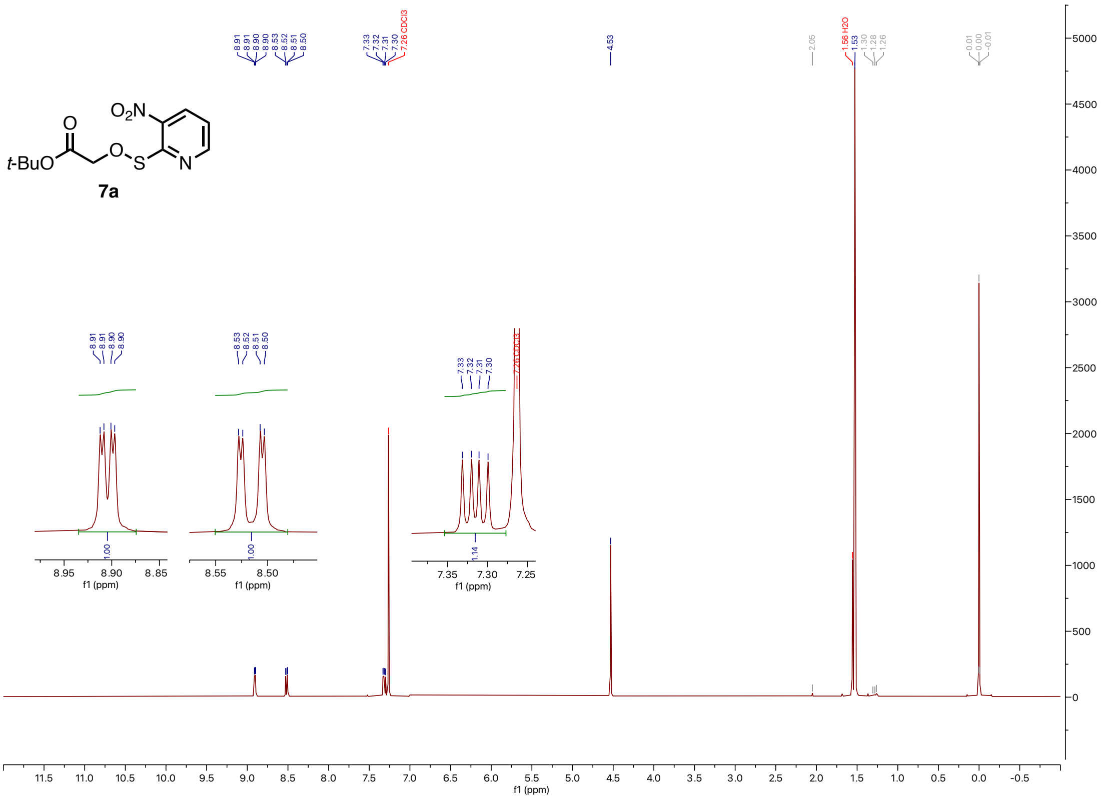

^{13}C NMR (100 MHz, $CDCl_3$)

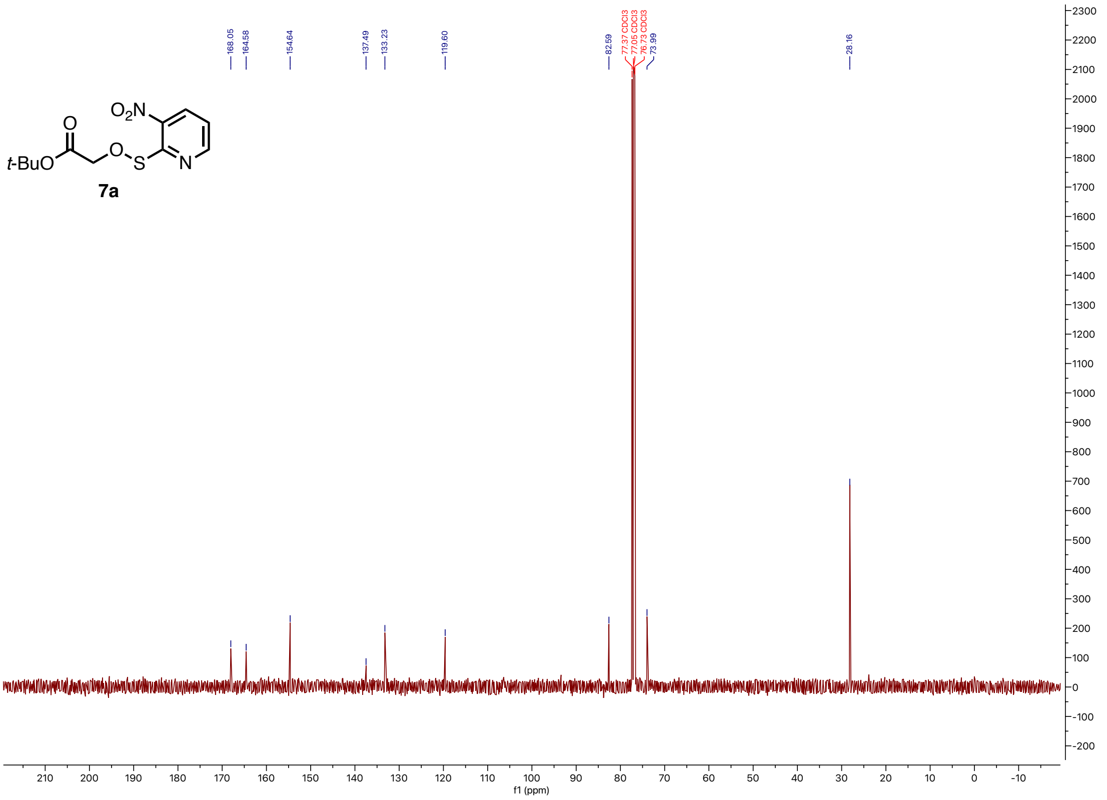

^{1}H NMR (400 MHz, $CDCl_3$)

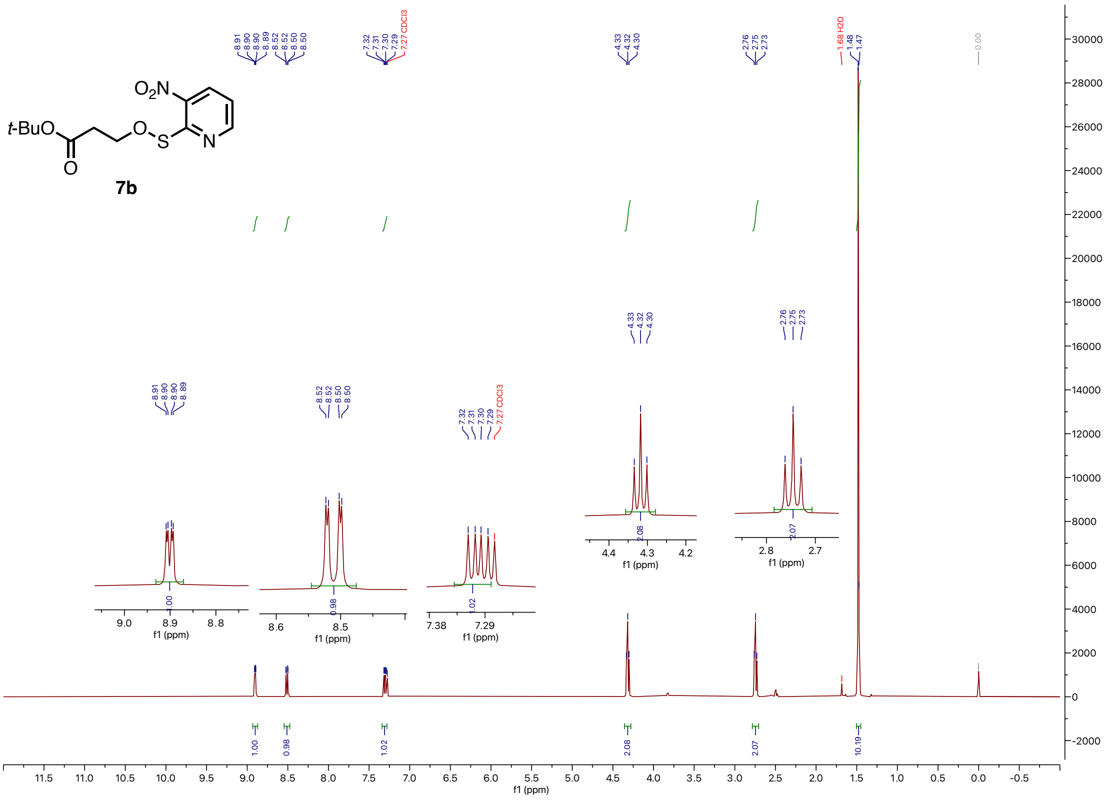

^{13}C NMR (100 MHz, $CDCl_3$)

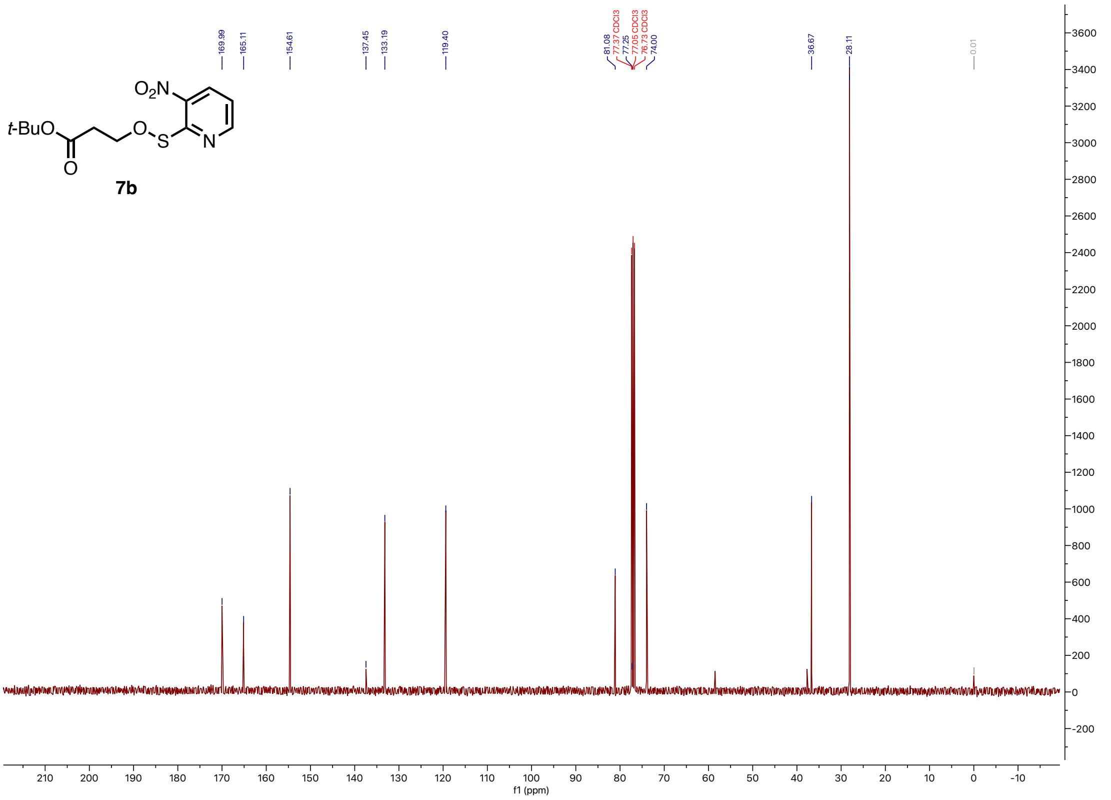

^{1}H NMR (400 MHz, $CDCl_3$)

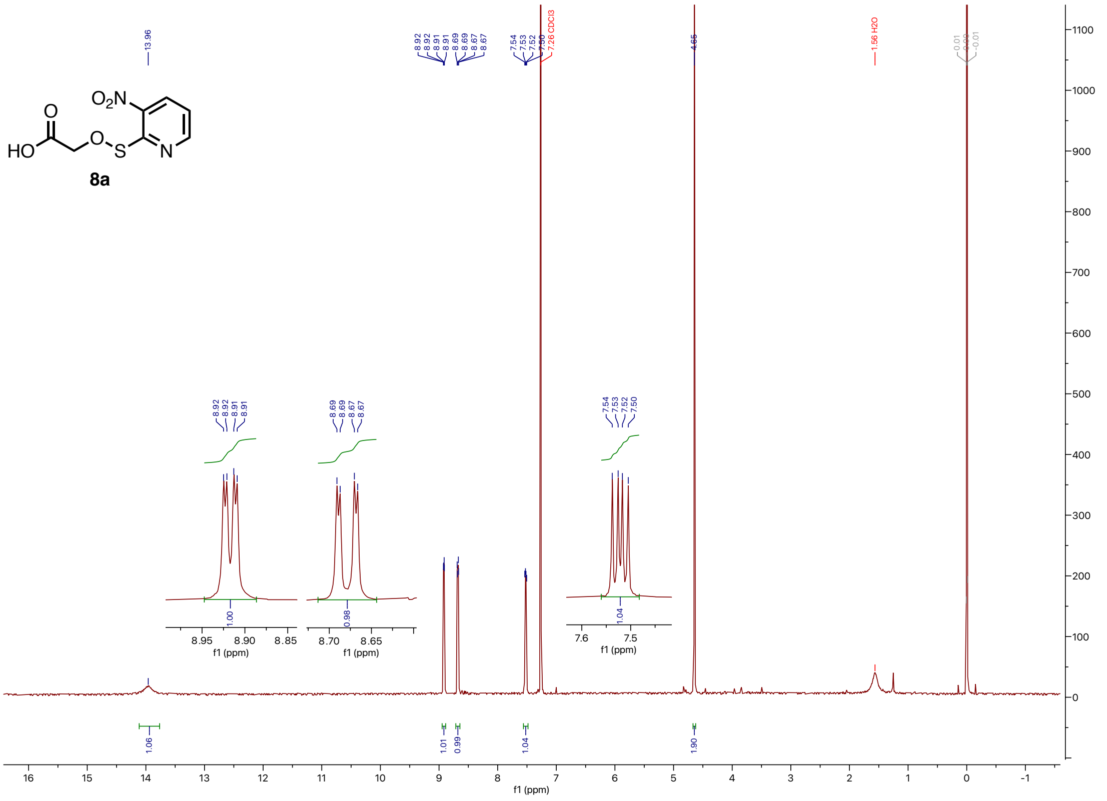

^{13}C NMR (100 MHz, CD_3OD)

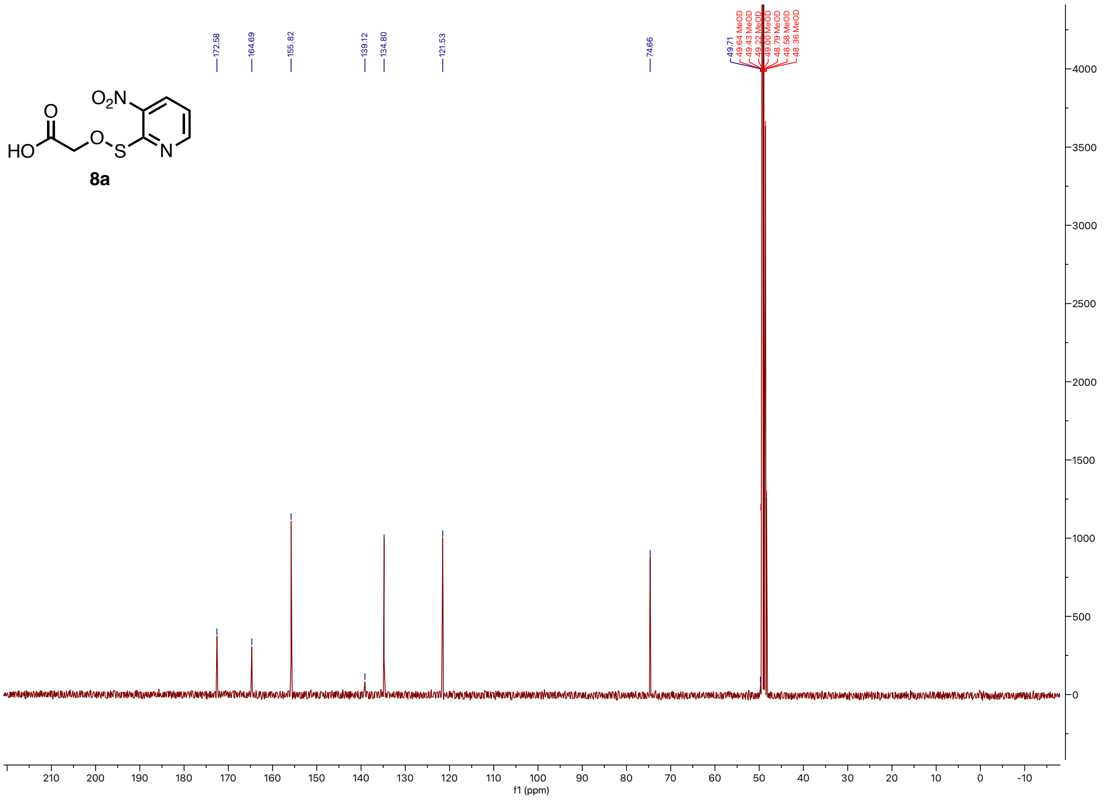

^{1}H NMR (400 MHz, $CDCl_3$)

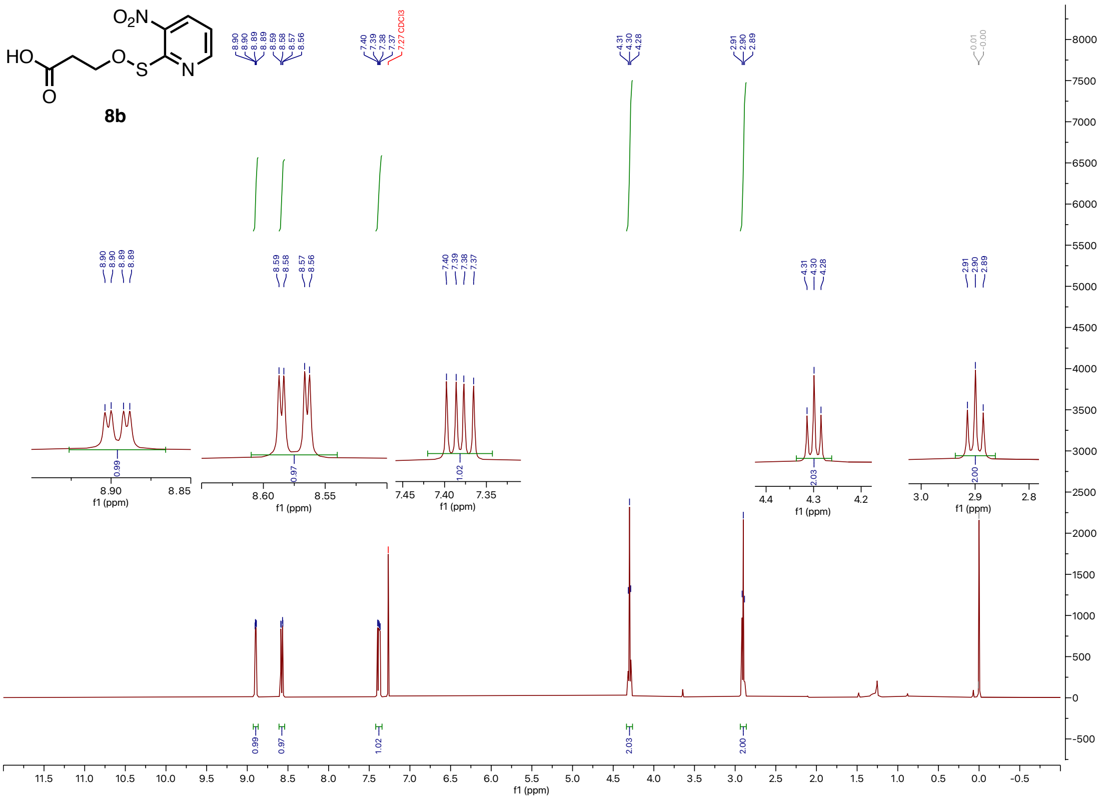

^{13}C NMR (100 MHz, $CDCl_3$)

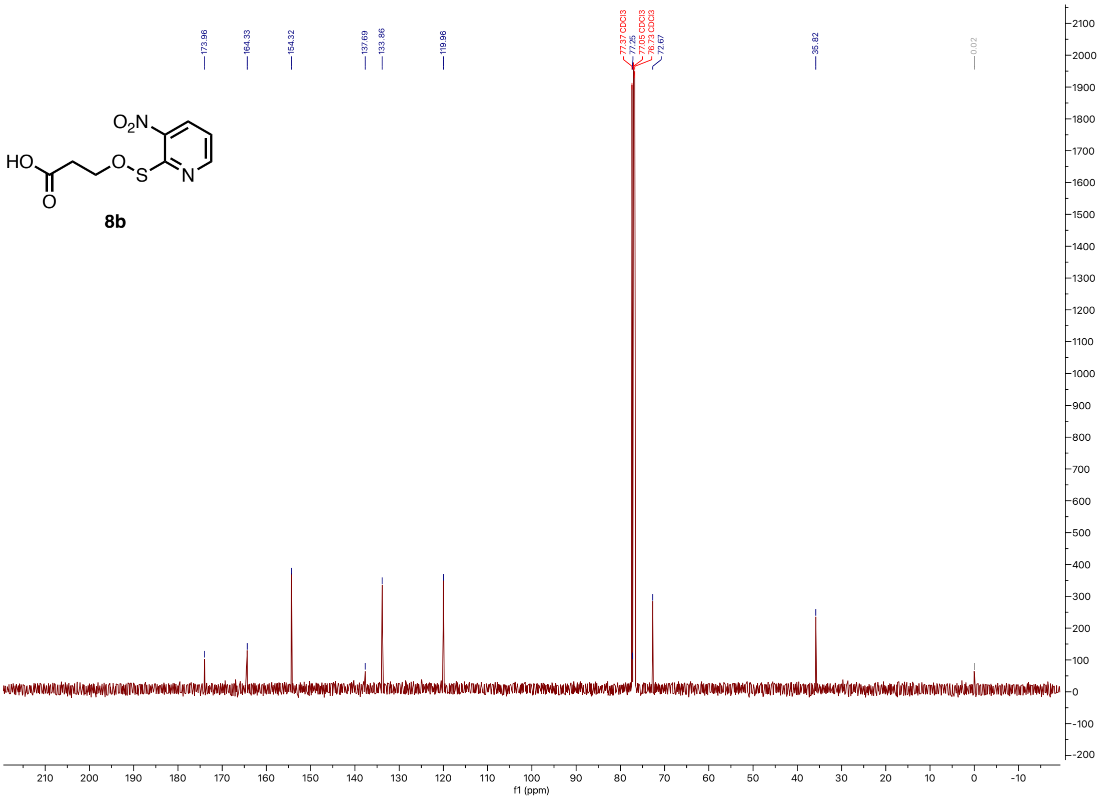

^1H NMR (400 MHz, CD_3OD)

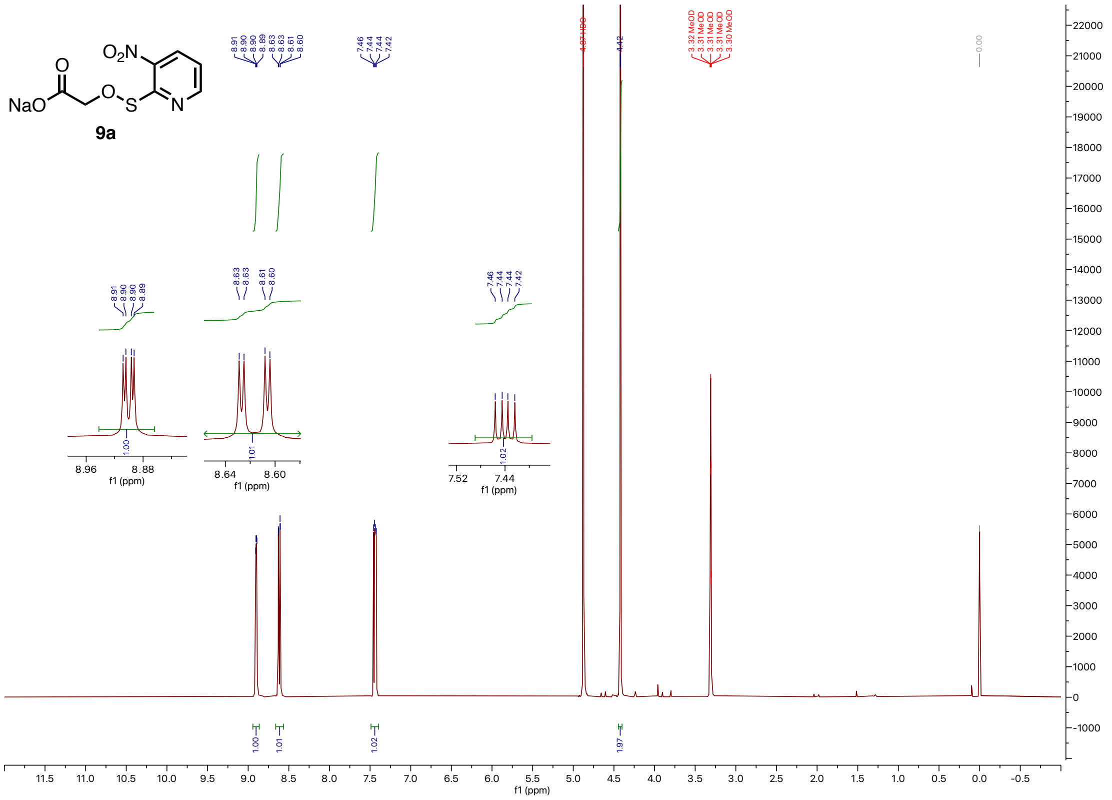

^{13}C NMR (100 MHz, CD_3OD)

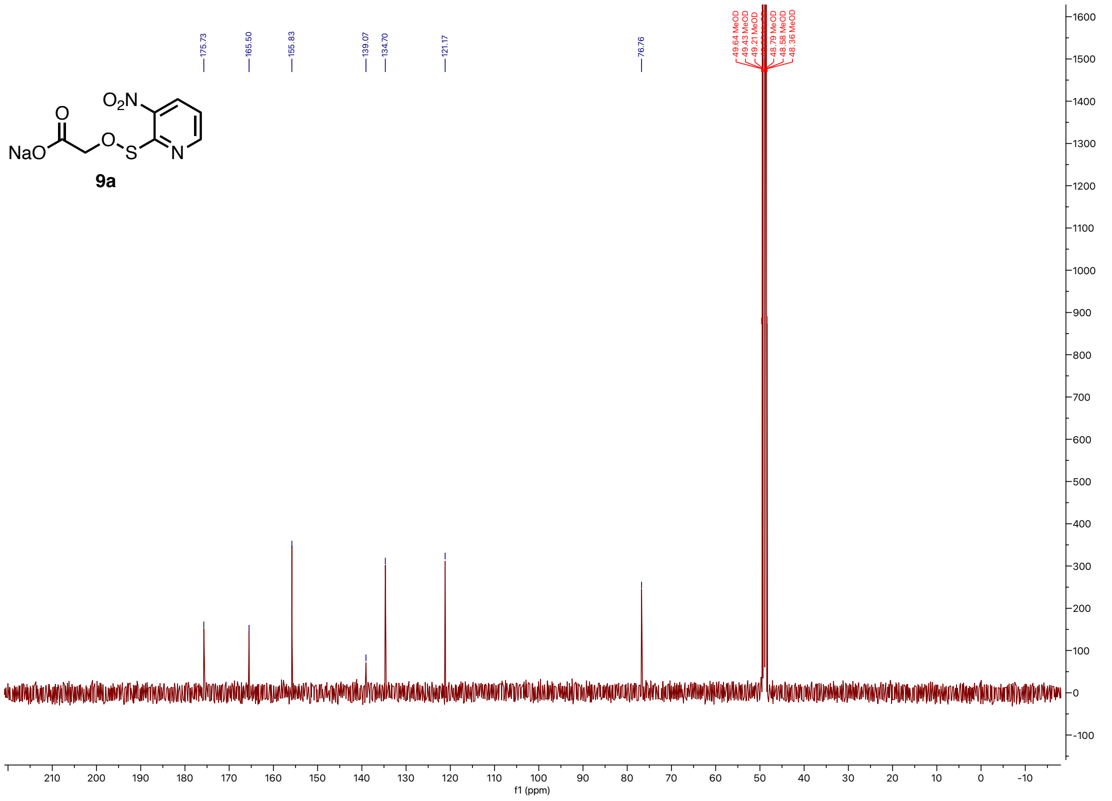

^{1}H NMR (400 MHz, DMSO-*d*6)

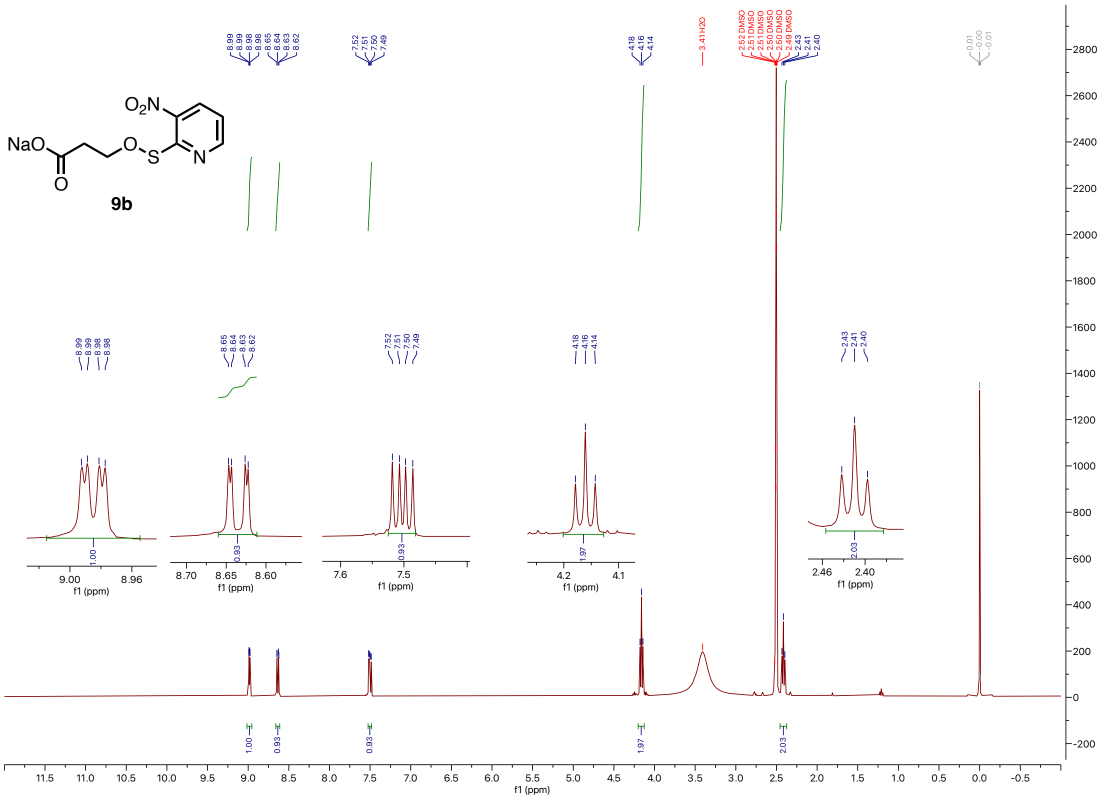

^{13}C NMR (100 MHz, D_2O)

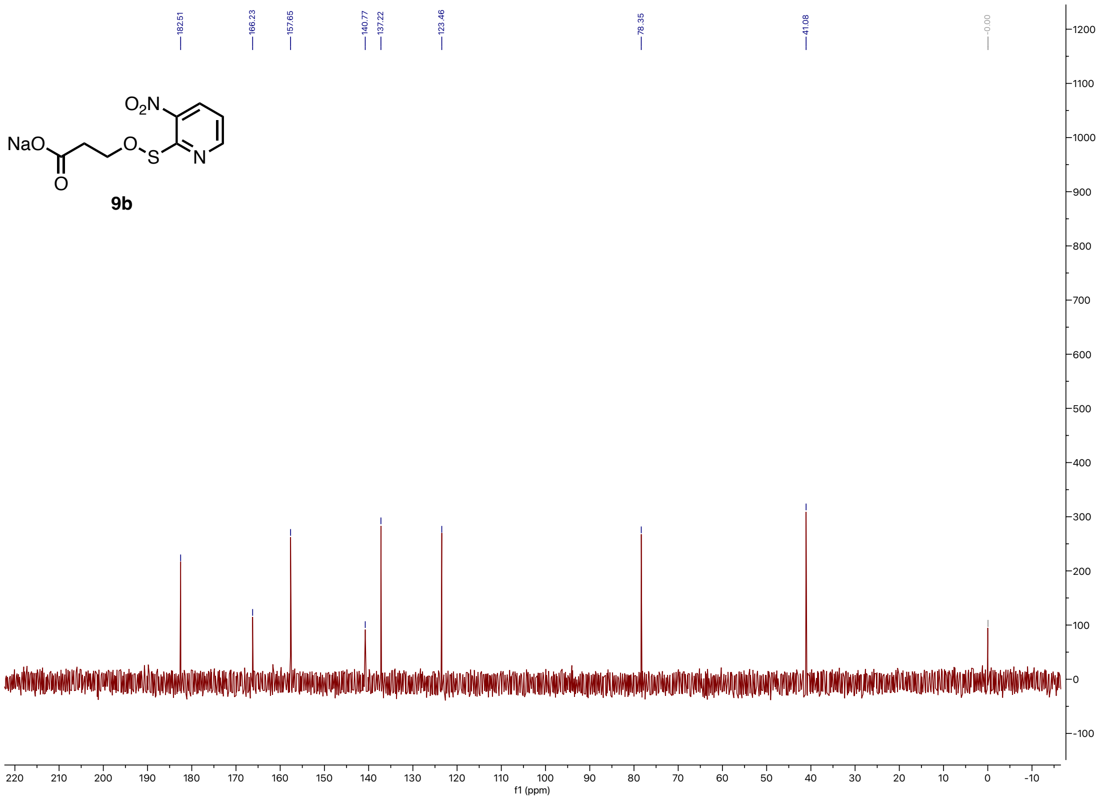

^{1}H NMR (400 MHz, CD_3OD)

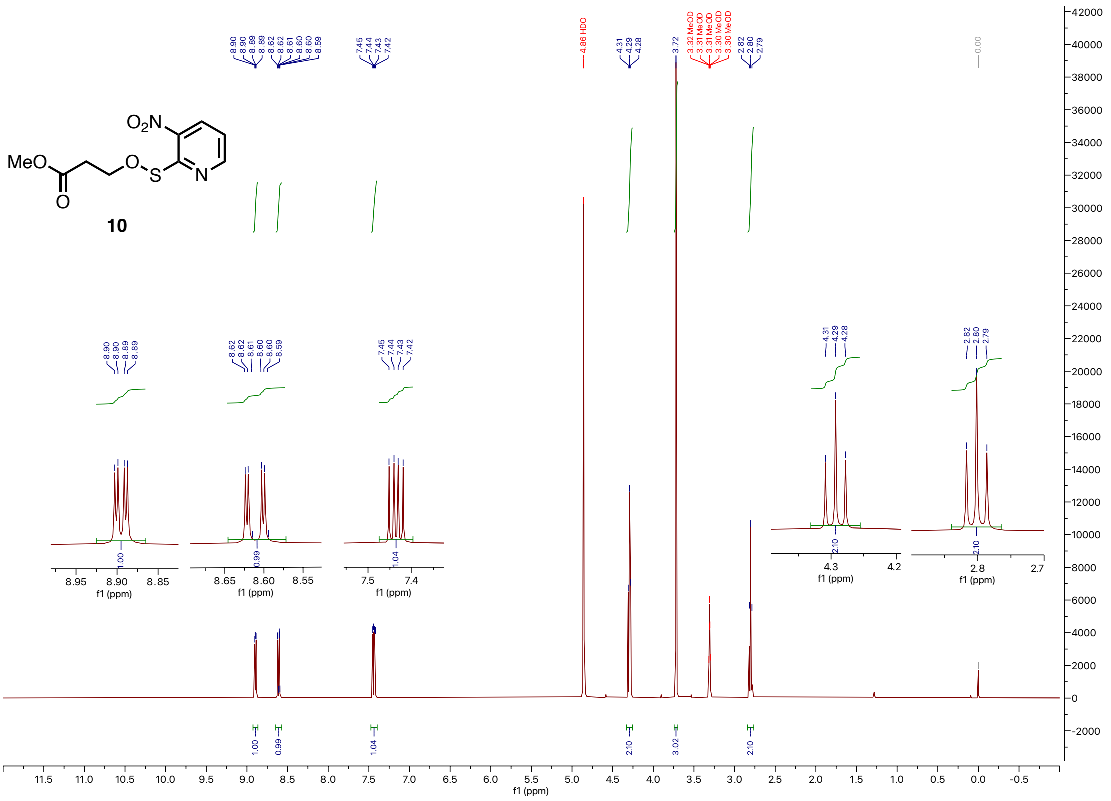

^{13}C NMR (100 MHz, CD_3OD)

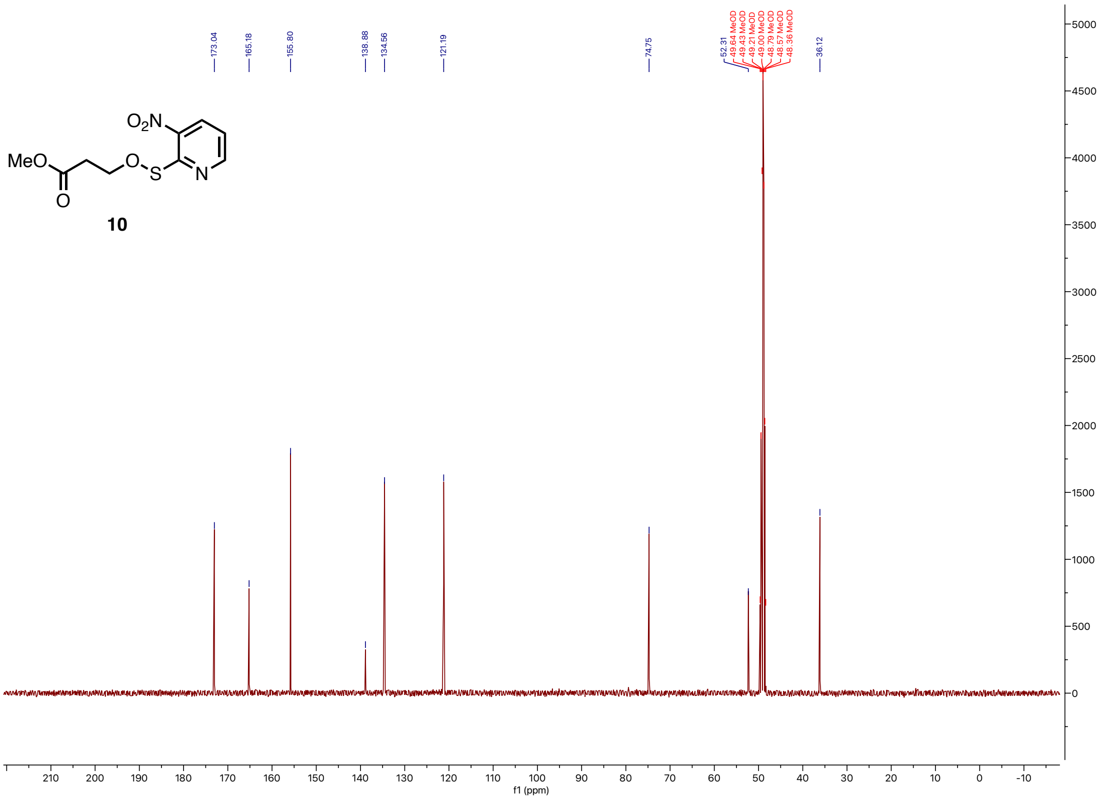

9. References

1. a) K. Muguruma, F. Yakushiji, R. Kawamata, D. Akiyama, R. Arima, T. Shirasaka, Y. Kikkawa, A. Taguchi, K. Takayama, T. Fukuhara, T. Watabe, Y. Ito, Y. Hayashi, *Bioconjug. Chem.* **2016**, *27*, 1606; b) K. Muguruma, T. Shirasaka, D. Akiyama, K. Fukumoto, A. Taguchi, K. Takayama, A. Taniguchi, Y. Hayashi, *Angew. Chem. Int. Ed.* **2018**, *57*, 2170; c) Y. Cui, A. Taguchi, K. Kobayashi, H. Shida, K. Takayama, A. Taniguchi, Y. Hayashi, *Org. Biomol. Chem.* **2020**, *18*, 7094.
2. a) A. Taguchi, K. Fukumoto, Y. Asahina, A. Kajiyama, S. Shimura, K. Hamada, K. Takayama, F. Yakushiji, H. Hojo, Y. Hayashi, *Org. Biomol. Chem.* **2015**, *13*, 3186; b) A. Taguchi, K. Kobayashi, Y. Cui, K. Takayama, A. Taniguchi, Y. Hayashi, *J. Org. Chem.* **2020**, *85*, 1495; c) H. Shida, A. Taguchi, S. Konno, K. Takayama, A. Taniguchi, Y. Hayashi, *Chem. Pharm. Bull.* **2023**, *71*, 435.
3. A. Taguchi, K. Kobayashi, A. Kotani, K. Muguruma, M. Kobayashi, K. Fukumoto, K. Takayama, H. Hakamata, Y. Hayashi, *Chem. Eur. J.*, 2017, **23**, 8262.
4. a) F. Yakushiji, H. Tanaka, K. Muguruma, T. Iwahashi, Y. Yamazaki, Y. Hayashi, *Chem. Eur. J.* **2011**, *17*, 12587; b) F. Yakushiji, K. Muguruma, Y. Hayashi, T. Shirasaka, R. Kawamata, H. Tanaka, Y. Yoshiwaka, A. Taguchi, K. Takayama, Y. Hayashi, *Bioorg. Med. Chem.* **2017**, *25*, 3623.
5. a) J. B. Blanco-Canosa, P. E. Dawson, *Angew. Chem. Int. Ed.* **2008**, *47*, 6851; b) J. Spengler, J. B. Blanco-Canosa, L. Forni, F. Albericio, *Org. Lett.* **2018**, *20*, 4306.
6. K. Sakamoto, S. Tsuda, H. Nishio, T. Yoshiya, *Chem. Commun.* **2017**, *53*, 12236.
